# Corrosion inhibition evaluation of *Bauhinia purpurea* extract mediated ZnO nanoparticles for carbon steel in acidic environment

**DOI:** 10.1186/s11671-026-04826-w

**Published:** 2026-08-21

**Authors:** O. Swathy, S. Shanmuga Priya, Meghana K. Navada, Lavanya Mulky

**Affiliations:** Manipal Institute of Technology, Manipal Academy of Higher Education, Manipal, Karnataka 576104 India

**Keywords:** Corrosion, Corrosion inhibitors, Nanomaterials, Green synthesis, ZnO nanoparticles, Carbon steel

## Abstract

ZnO-NPs were synthesised using *Bauhinia purpurea* leave*s* and explored as green nano corrosion inhibitors for carbon steel in 1M hydrochloric acid. Comprehensive characterization of the synthesized ZnO-NPs was characterized using Fourier transform infrared (FTIR) spectroscopy, ultraviolet–visible (UV–vis) spectroscopy, scanning electron microscopy (SEM), and energy-dispersive X-ray spectroscopy (EDS). Electrochemical methods, including potentiodynamic polarization (PDP) and electrochemical impedance spectroscopy (EIS), were performed to evaluate the corrosion inhibition efficiency at varying nanoparticle concentrations. Surface analyses through SEM, XPS, FTIR, and EDS confirmed the ZnO-NPs adsorption on the CS surface, validating the proposed inhibition mechanism. ZnO-NPs synthesized by sol–gel and co-precipitation methods exhibited maximum inhibition efficiencies of 94% and 97% at 100 ppm and 303 K, respectively, as determined by PDP. Consistently, EIS analysis demonstrated superior inhibition performance, with efficiencies of 99% (co-precipitation) and 98% (sol–gel) at 100 ppm. The findings confirm that green-synthesised ZnO-NPs act as effective, eco-friendly inhibitors by forming a protective adsorbed layer on the steel surface, thereby mitigating acid-induced corrosion.

## Introduction

Carbon steel (CS) is a strategically significant metallic material and is widely utilised as an advanced metal in pickling, electroplating, chemical equipment, and other industrial sectors due to its outstanding weldability, thermal conductivity, and ductility; however, it also suffers from corrosion problems. Although corrosion is not entirely preventable, its pace can be managed with the addition of an appropriate inhibitor. They are widely employed for acid cleaning, oil well stimulation, and the removal of localised deposits. HCl is the most often used medium in corrosion studies of these alloys. Corrosion is a major global issue that has affected many industries. The corrosion of CS brought on by dissolved CS alloys carbon dioxide in the form of carbonic acid, H_2_CO_3_ indicates that the use of carbon in CS is limited, particularly in companies that generate large amounts of CO_2_. The most prevalent forms of corrosion in oil fields are CO_2_, H_2_S, and corrosion brought on by dissolved oxygen in water [1]. Corrosion, which usually occurs abruptly and under strong mechanical and thermal forces, might cause a promising new technology to fail. However, it is most commonly experienced in nuclear reactors, turbines, combustion engines, automobiles, petrochemical plants, and hydraulic and power aggregates. Therefore, it is evident that corrosion research and development would reduce corrosion damage and help the country save money in all technological fields [2]. CS has been designed to be resistant to corrosion using a range of methods, such as coating, inhibitor, and cathodic or anodic protection. Among the several corrosion management techniques, using inhibitors is the most accessible and reasonably priced way to prolong the life of metal. A corrosion inhibitor can reduce the liquid or gas rate of corrosion [3–5]. However, the problem of CS corrosion necessitates attention and investigation, particularly in situations with HCl acid.

**Fig. 1:**
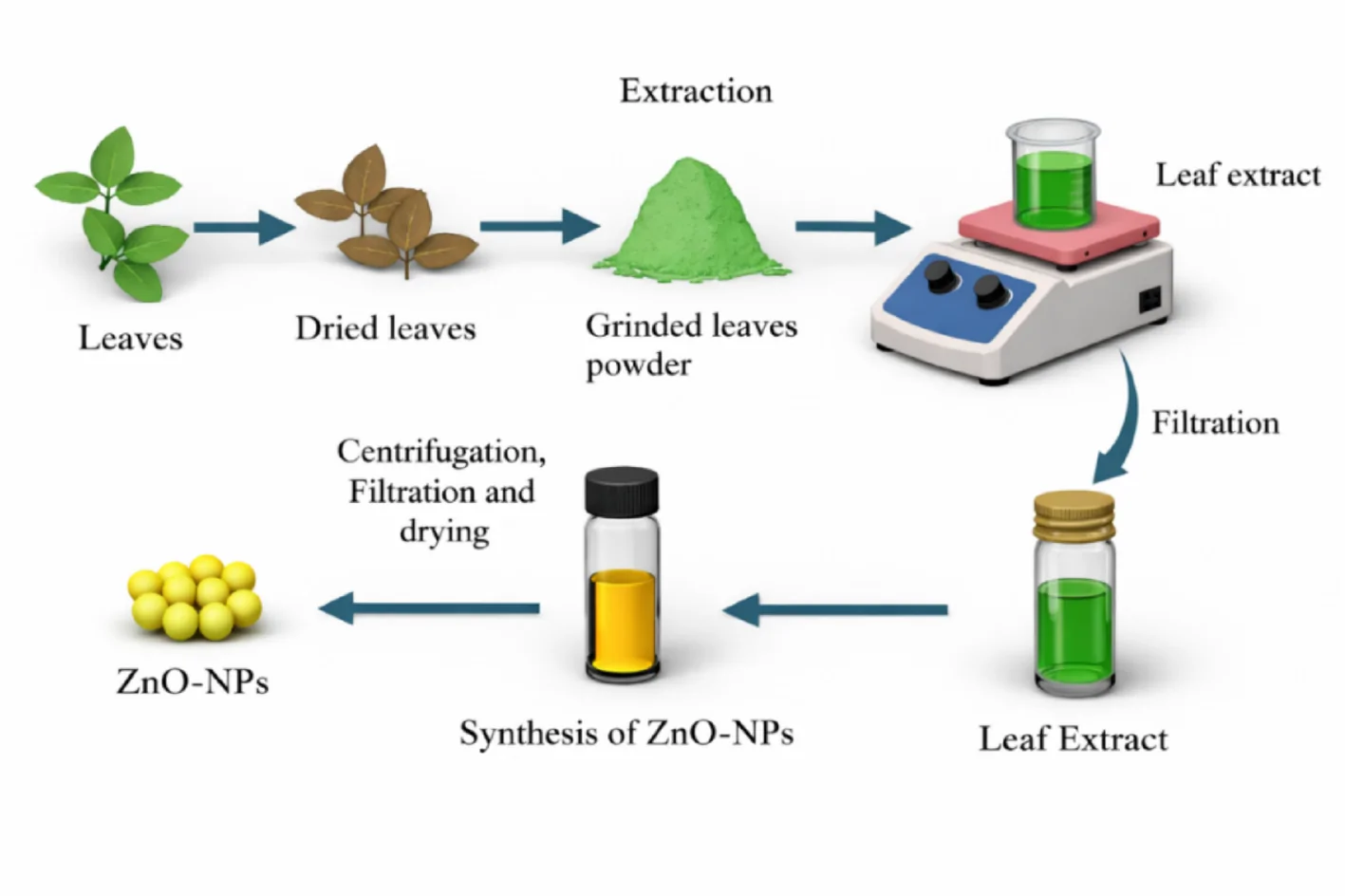
Schematic representation of green synthesis of ZnO-NPs.

Organic corrosion inhibitors are gradually replacing inorganic corrosion inhibitors with significant toxicity as environmental protection and sustainable development become more widely recognised [6]. Typically, corrosion inhibitors are heterocyclic organic compounds with high electron density heteroatoms like N, O, S and P as well as double or triple bonds [7–9]. There is growing interest in generating efficient, economical, and ecologically friendly substitutes [10, 11]. Sourced from renewable natural resources, green inhibitors are non-toxic, biodegradable, and environmentally safe [12]. Their mode of action involves absorbing onto metal surfaces to generate layers of protection, thereby preventing corrosion. Nanoparticles (NPs), with an average size ranging from 1 to 100 nanometres, are considered more effective corrosion inhibitors because of their unique physicochemical properties [13, 14]. Plants are considered the best source for green synthesis of NPs because of their availability, cost-effectiveness, and efficiency. The presence of secondary metabolites, such as tannins, flavonoids, saponins, alkaloids, and steroids, acts as reducing and capping agents during synthesis. These molecules have electron-rich hetero negative atoms like oxygen, nitrogen and pi bonds so they easily reduce the metal precursor into NPs producing monodispersed green NPs with precise size and shape, overcoming the challenge of nanocontainment. The general procedure of green synthesis of NPs has been illustrated in Fig. 1 [15]. This research focuses on integrating green-synthesized ZnO-NPs enhance corrosion inhibition efficiency in CS. However, an abundance of natural substances is suitable for CS as corrosion inhibitors. The adsorption of organic molecules on the metal surface is responsible for their inhibitory effect. Heteroatoms like N, O, and S have a larger electron density and their presence in heterogeneous organic compounds often increases the protection ratio against corrosion. These atoms are therefore regarded as the active sites of the adsorption on the metal [16].

In the context of green nano inhibitors recently, multiple reports show that green synthesised ZnO-NPs function as sustainable corrosion inhibitors for various steel grades exposed to acidic and chloride-containing electrolytes, compared to conventional ZnO-NPs. *Lagerstroemia speciosa extract* mediated ZnO-NPs showed enhanced inhibition efficiency of 94%, in 1 M HCl for CS [17]. Beddiar et al. showed that Daucus *crinitus* extract-mediated ZnO-NPs afford 91.20% protection to CS in HCl [18]. Similarly, Dalmaz et al. demonstrated that *Laurus nobilis* leaf-based ZnO-NPs reach 97.1% inhibition efficiency for St-37 steel in NaCl medium [19]. In another study of ZnO-NPs synthesized from *Plectranthus amboinicus* leaves deliver 78.54% inhibition efficiency for X60 steel in acidic solution [20]. Elebo et al. reported that *Opuntia fragalis leaf*-derived ZnO-NPs achieve 77.31% efficiency for mild steel in HCl [21]. Al-Dahiri et al., Al-Senani et al., and Ifeanyichukwu et al. further showed that *Myrrh*-, *Convolvulus*-, and *Punica granatum-*based ZnO-NPs provide 92%, 91%, and 99.9% inhibition, respectively, highlighting the ability of green-synthesized ZnO-NPs to form stable, adherent, and electrochemically resistant films on steel surfaces in HCl medium [22–24].

The current work is deemed significant because of its cost-effective inhibitory properties, industrial application, and environmental friendliness. A plant belonging to the *Caesalpiniaceae* family, *Bauhinia purpurea* is widespread throughout India and throughout Asia. *Bauhinia purpurea leaves* were screened for phytochemicals and found to include flavonoids, triterpenes, saponins, and steroids, but no tannins or alkaloids. The phytochemicals present in *Bauhinia purpurea*, particularly those containing hydroxyl and carbonyl functional groups, facilitate the reduction of Zn^2+^ ions and serve as reducing, capping, and stabilizing agents during the green synthesis of ZnO-NPs. Furthermore, these bioactive compounds contribute to corrosion inhibition by adsorbing onto the metal surface and forming a stable protective barrier that limits further corrosive attack. The novelty of this work lies in the comparative evaluation of ZnO-NPs synthesized via the sol–gel and co-precipitation methods for corrosion inhibition of CS in 1 M HCl. Furthermore, the influence of the synthesis route on nanoparticle characteristics, adsorption behavior, and corrosion protection performance was systematically investigated using electrochemical (PDP and EIS) and surface characterization (SEM, FTIR, and EDS) techniques, providing new insights into the relationship between the synthesis method and corrosion inhibition efficiency [25–27].

## Materials and methods

### Materials

Hydrochloric acid (37% HCl), CS block (1 × 1 × 1 cm), dried leaves powder. Without any additional chemical processing, all materials are used straight away. Analytical-grade Ethanol, zinc nitrate hexahydrate (Zn (NO_3_)_2_·6 H_2_O), of analytical grade reagents were procured from Sigma-Aldrich laboratory in Bangalore. The solvents used in the research study were procured from the same suppliers and used without further purification.

### Test coupons

Corrosion test was performed on cylindrical CS coupon with a surface area of 1cm^2^ and diameter of 10 mm. The CS surface mounted with cold-setting resin and polished using silicon carbide emery papers up to grade 2000 and disc-polished until a mirror shine was obtained on the surface. The coupons are cleaned with acetone followed by washing with double-distilled water, and dried. Further corrosion study could be performed on the CS. Table 1 provides the composition of CS.

**Table 1:** Elemental composition of the CS specimen used in this study, showing the percentage content of major and minor alloying elements.

Fe	C	Mn	Si	S	*P*	Cr	Ni	Mo
98.6	0.20	0.69	0.20	0.02	0.03	0.09	0.06	0.01

### Preparation of medium

Analytical grade, 37% HCl was diluted with distilled water to make the test solution of 1 M HCl.

### Preparation of plant extract

The *Bauhinia purpurea* leaves are gathered and repeatedly cleaned with distilled water to remove contaminants. The leaves were shade-dried and pulverised using a juicer. 100 mL of distilled water was mixed with 5 g of *Bauhinia purpurea* powder, and the mixture was heated to 60°C for 30 min. In order to prevent the breakdown of heat-sensitive molecules, the solution was allowed to naturally cool to room temperature following the extraction. Soon after cooling, the solution was filtered through Whatman filter paper and kept in a fridge for further use [28].

### Synthesis of ZnO-NPs

#### Co-precipitation method

5 g of Zn (NO_3_)_2_.6H_2_O was completely mixed in 150 mL of distilled water, the mixture was continuously swirled and gradually supplemented with a 50 mL aqueous extract of *Bauhinia purpurea*. The colour of solution changed noticeably from translucent to pale yellow, indicating successful NPs formation. The sample is allowed to cool at room temperature. The precipitated particles then separated using a filtration process and then washed with ethanol. For total purification, five centrifugations at 10,000 rpm were carried out to eliminate pollutants. The precipitates were calcined for eight hours at 100 °C and annealed at 300 °C [29].

#### Sol–gel method

50 ml of *Bauhinia purpurea* extract was stirred with a stirrer-heater. 5 g of Zn(NO_3_)_2_.6H_2_O dissolved in distilled water(100 mL) were added to the solution at room temperature. NaOH is added dropwise to the solution until pH becomes 12 forming a white gel, which indicates the formation of ZnO-NPs and kept stirring for 1 h. Following centrifugation, the particles collected and oven-dried for a few hours at 70 °C. After annealing, the white powder made of ZnO-NPs was utilized for additional characterisation [30]. ZnO-NPs are produced via green synthesis using green method is shown in Fig. 1.

### Materials characterizations

Several characterization tools have been used in the study to provide insight into the characteristics of ZnO-NPs. Using X-ray powder diffraction (XRD) on a Bruker AXS-D8 diffractometer-USA, the crystal shape of the produced ZnO-NPs were examined. Operating at 35 kV, the device used Cu-Ka radiation with a wavelength of 1.54 Å to scan within the 2q range of 30°–80° at a speed of 1 s per step. A Shimadzu-Japan ultraviolet–visible (UV–vis) spectrophotometer (UV-2600) was used to view the absorbance spectra and bandgap of ZnO in the wavelength range of 300–600 nm. In order to perform an absorbance analysis for UV-Vis spectroscopy, 1 mg of ZnO-NPs was dissolved in water to create a uniform suspension.

The functional groups found in ZnO, and the extract were identified using a Shimadzu-Japan Fourier transform infrared (FTIR-8400) spectrometer, which has a scanning range of 500–4000 cm^− 1^. Spectroscopic-grade potassium bromide (KBr) was completely combined with a tiny amount of powdered sample in a dry atmosphere before being compressed into a transparent pellet using a hydraulic press for FT-IR analysis. In contrast, two to three drops of the extract were employed for the analysis. Thermo Scientific AAS quantified dissolved iron in test solutions post-corrosion. Optimized flame conditions supported the analysis. Standard iron solutions calibrated the instrument. Samples underwent dilution and filtration beforehand. This ensured precise, reproducible data.

The scanning electron microscope (SEM, Supra-55VP) of Carl Zeiss, Germany, was used for morphological analysis and particle size determination. Energy dispersive X-ray spectrum (EDX) and selected area diffraction (SAED) pattern were also used. A suspension containing 2 mg of ZnO -NPs was produced for SEM investigation, and a few drops were added to a conductive substrate before being dried to ensure optimal adhesion and imaging. Thermo Scientific K-Alpha system performed XPS measurements. It featured a 180° double-focusing hemispherical analyser. A 128-channel detector captured signals. Monochromatic Al Kα X-ray source used micro-focus. Spot size ranged 50–400 μm. Dual-beam system neutralized charge. EX06 ion gun operated from 200 eV to 2 keV. Modules enabled ARXPS, work function analysis, and vacuum transfer.

### Corrosion rate measurement techniques

The electrochemical workstation CH instrument USA model 604 D series with beta software was used to conduct the potentiodynamic polarization investigations. Three-electrode Pyrex glass cells were used for the analysis, which was carried out at three distinct concentrations (50 ppm, 75 ppm, and 100 ppm). Platinum served as the auxiliary electrode, CS served as the working electrode, and calomel served as the reference electrode. To maintain a steady temperature (303 K) throughout the whole experiment, the cell was submerged in a thermostat.

#### Potentiodynamic polarization (PDP) measurement

The test coupon was initially submerged in a caustic for about 1200 s in order to measure the open-circuit potential (OCP). At a scan rate of 1 mV/s, the specimen was polarised for PDP from − 250 mV cathodically to + 250 mV anodically.

#### Electrochemical impedance spectroscopy (EIS)

Impedance measurements are performed at the OCP using an AC signal with an amplitude of 10 mV in the frequency range of 100 kHz to 0.01 Hz. At least three experiments were carried out in each case, and the average of the most reliable values are taken.

### Surface morphology studies

An analytical SEM was used to examine the CS surface morphology with and without an inhibitor. For three days, CS is submerged in a 1M HCl solution. The SEM pictures are then taken after it has been cleaned with distilled water and dried. Additionally, EDX is employed to determine elemental composition. The surface chemical composition and inhibition mechanism were investigated using XPS.

## Result and discussion

### UV–vis spectrum analysis

**Fig. 2:**
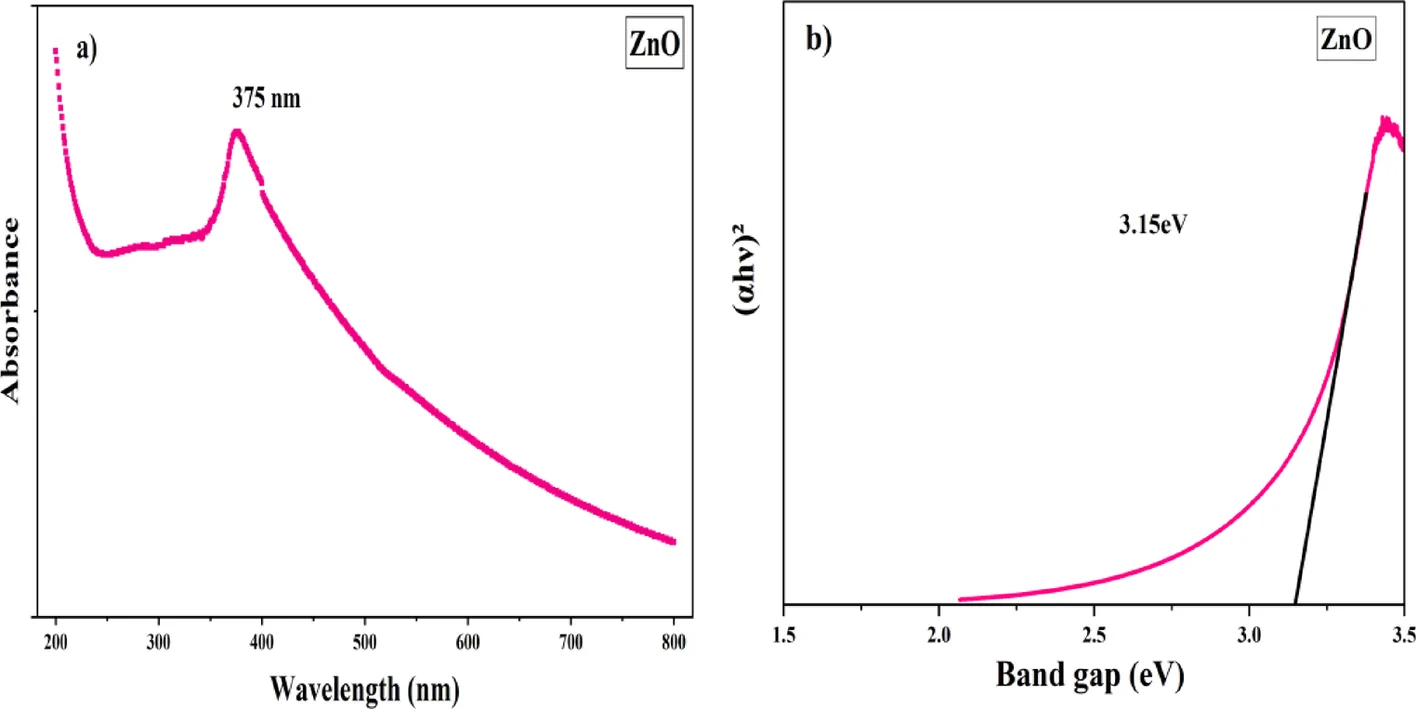
a) UV–vis spectrum of ZnO-NPs prepared using *Bauhinia purpurea extract *via co-precipitation method **b)** Tauc plot of the same UV–vis spectrum.

**Fig. 3:**
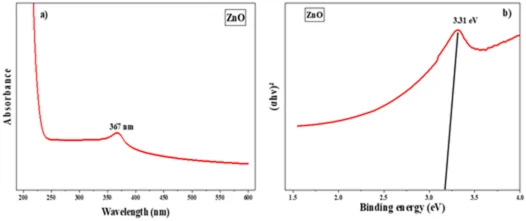
UV–vis spectrum of ZnO-NPs prepared using *Bauhinia purpurea leaf extract* via sol–gel method **b)** Tauc plot of the same UV–vis spectrum.

The optical behavior of the ZnO-NPs was examined by Ultraviolet–Visible (UV–Vis) spectroscopy. The combined spectra of co-precipitation-mediated ZnO and sol–gel mediated ZnO-NPs, plotted as absorbance versus wavelength (nm), are shown in the Fig. 2 and 3. Both systems display a strong absorption feature in the 360–380 nm region, which lies within the typical ZnO-NPs absorption region 300–380 nm, thereby verifying the formation of ZnO-NPs. It is visible that the maximum absorbance of ZnO lies in the visible region due to electron trapping [31].

The optical band gap was evaluated using the Tauc Eq. 1 by plotting $(\alpha h\nu )^{2}$ against $\:h\nu\:$, where $\:\alpha\:$ denotes the absorption coefficient and $\:h\nu\:$ represents the photon energy (eV), as illustrated in Fig. 2. The Tauc plot is obtained, utilizing the given equation for direct bandgap materials by plotting $(\alpha h\nu )^{2}$ versus photon energy $\:h\nu\:$ with linear extrapolation.

1 $$\:(\alpha\:h\nu\:{)}^{2}=B\left(h\nu\:-{E}_{g}\right)$$

Extrapolation of the linear segment of the Tauc plot to the energy axis yielded direct band gap values of 3.15 eV for co-precipitation-mediated ZnO-NPs and 3.31 eV for sol–gel mediated ZnO-NPs. This value falls in the bulk ZnO (3–3.37 eV), attributable to quantum confinement effects where smaller particles widen bandgap, alongside synthesis method, lattice strain, morphology, grain size, defects, and surface roughness. Accordingly, the relatively larger ZnO-NPs exhibit a lower band gap than the smaller sol–gel ZnO-NPs [32]. The UV–Vis absorption peak in ZnO-NPs originates from intrinsic band-gap transitions, where photons promote electrons from the valence band to the conduction band of ZnO. The slight blue shift to 367 nm for the co-precipitation sample shows a marginally larger effective band gap, which can result from smaller crystallite size, different morphology, or higher lattice strain or defect density compared with the sol–gel ZnO-NPs [33–35].

According to reports, the possibility of increased corrosion activity of the NPs is indicated by a narrower band gap, which is especially noticeable in the corrosion inhibition. This phenomenon results from the smaller band gap, which makes it easier to excite electrons from the valence band to the conduction band [36].

### FT-IR analysis

**Fig. 4:**
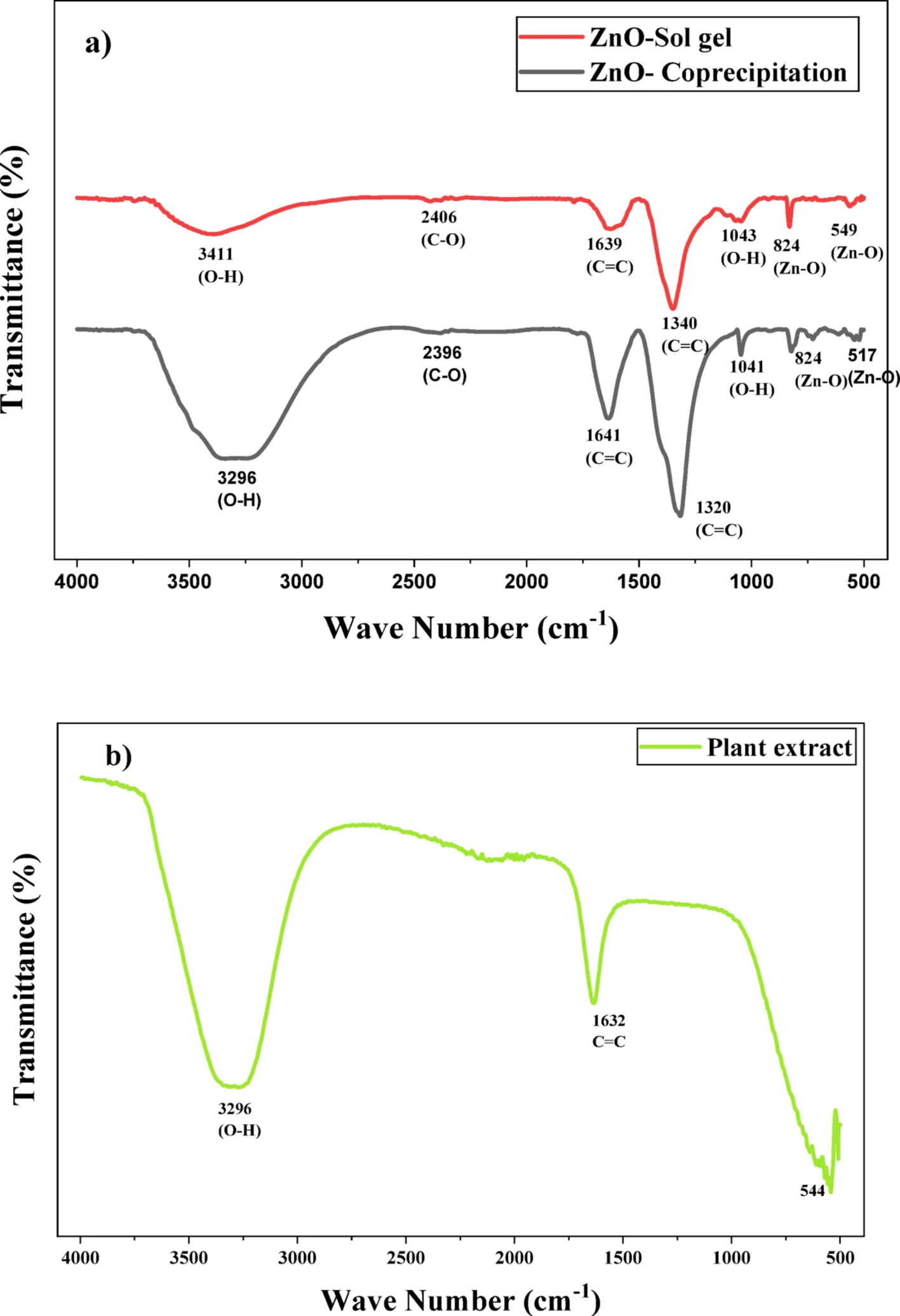
FTIR spectra of a) ZnO-NPs synthesised from *Bauhinia purpurea* extract **b)** FTIR spectra of *Bauhinia purpurea* extract.

The FT-IR spectra of ZnO samples and plant extract are captured between 400 and 4500 cm^− 1^, as shown in Fig. 4. ZnO-NPs showed multiple unique peaks compared to the extract alone. The spectrum of the plant extract reveals a broad absorption band at 3266 cm⁻¹, which is attributed to O–H stretching vibrations of hydroxyl groups, suggesting the presence of alcoholic and phenolic compounds. The peak observed at 1632 cm^− 1^ corresponds to C=O stretching vibrations of carbonyl groups, which may also overlap with C=C stretching of aromatic structures, indicating the presence of flavonoids, tannins, or related phytochemicals. Additionally, the band at 594 cm^−1^ falls in the fingerprint region and can be associated with skeletal vibrations or C-X stretching, further confirming the complexity of functional groups present in the extract. The presence of these characteristic bands confirms that the extract contains bioactive molecules such as polyphenols and flavonoids, which can donate electrons or forming complexes, making them responsible for the observed antioxidant and corrosion inhibition activities. FTIR analysis was carried out to identify the functional groups of ZnO-NPs synthesised by green co-precipitation method. The presence of ZnO is confirmed by an FTIR plot, which usually displays a noticeable peak in the wavenumber range of 400–600 cm^−1^. The Zn-O stretching vibration is represented by this peak. O–H stretching vibrations are shown by the large peak at 3296 cm^−1^, which suggests the presence of hydroxyl groups from either surface hydroxyls on the NPs or adsorbed water molecules. C–O stretching vibrations are probably the cause of a distinct peak at 2368 cm^−1^, which may be connected to adsorbed CO_2_ or carbonyl groups. The peak at 1641 cm^−1^ signifies C=C stretching vibrations, which may arise from residual organic compounds, such as those derived from plant extracts used during synthesis or as stabilizing agents. Another C=C stretching peak at 1320 cm^−1^ suggests contributions from aromatic or other organic residues. Finally, the characteristic peaks at 824 cm^−1^ and 517 cm^−1^ are associated with Zn–O stretching vibrations, verifying the ZnO-NPs synthesis with the strong signal at 517 cm^−1^ being a definitive marker of the Zn–O bond. These observations collectively validate the successful synthesis and surface composition of ZnO-NPs [37, 38].

In the FTIR spectra of the ZnO-NPs, the presence of both ZnO and functional groups from *Bauhinia purpurea* is confirmed by the significant absorption lines. O–H stretching vibrations are responsible for the broad peak at 3411 cm^−1^, which is probably caused by water molecules or phytochemicals used in the green sol–gel production process. The peak at 1639 cm^−1^ describes C=C stretching vibrations, which may be the result of aromatic compounds in the extract, whereas the band at 2406 cm^−1^ may reflect adsorbed CO_2_ or C–O stretching. The C=C bending and C–O stretching modes may be represented by the additional peaks at 1340 cm^−1^ and 1043 cm^−1^, respectively. The successful synthesis of ZnO-NPs is confirmed by the assignment of significant absorption bands at 824 cm^−1^ and 549 cm^−1^ to Zn–O stretching vibrations [39, 40].

### XRD analysis

**Fig. 5:**
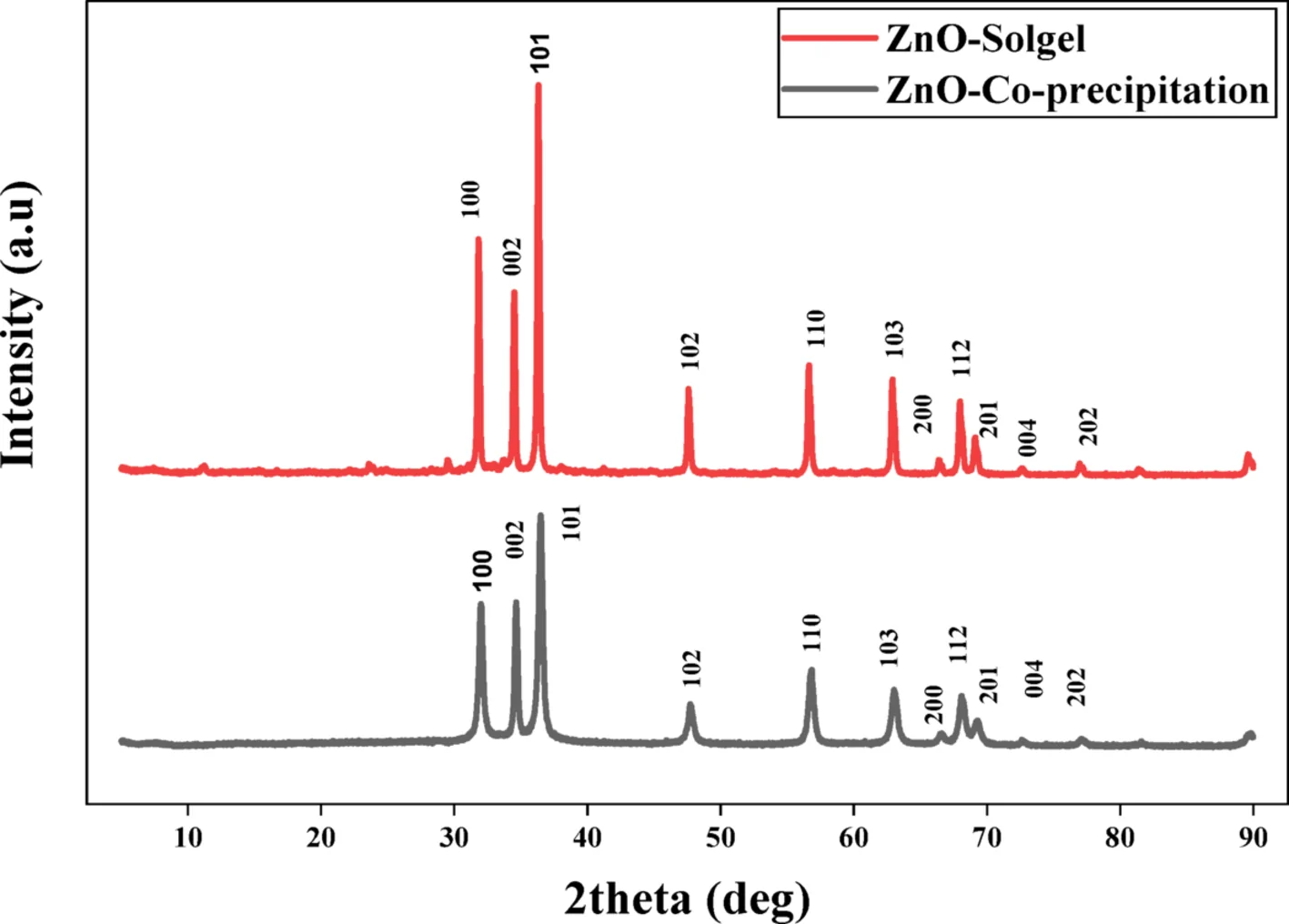
XRD pattern ZnO-NPs synthesised from different methods.

The diffraction patterns of biosynthesized ZnO-NPs were depicted in Fig. 5. XRD patterns of ZnO-NPs showed distinct peaks in the spectrum of 2*θ* values at 31.87°, 34.51°, 36.29°, 47.63°, 56.66°, 62.95°, 66.45°, 67.9°, 69.9°, 72.83° and 77.09° that correspond to the (100), (002), (101), (102), (110), (103), (200), (112), and (201), (004), (202) crystal planes. This confirms that the hexagonal wurtzite structure of ZnO was formed in accordance with JCPDS card no.36-1451. A high degree of crystallinity is indicated by the crisp and strong diffraction peaks, and the intense (101) reflection implies that this orientation is the favoured one. The green synthesis method used to create these ZnO-NPs generated using plant extracts, which serve as stabilizing and reducing agent, causes the extra peaks. The XRD pattern found is consistent with previous publications on green-synthesised ZnO-NPs [41].

The XRD pattern of synthesised ZnO-NPs demonstrates clear peaks at 2θ values 32.04°, 34.68°, 36.47°, 47.71°, 48.24°, 56.82°, 63.05°, 66.54°, 68.15° 69.17°, 72.66° and 77.26° that correspond to the (100), (002), (101), (102), (110), (103), (200), (112), and (201), (004), (202) planes. This suggests that the wurtzite crystal structure is the hexagonal phase (JCPDS card no.36-1451) [42]. Great crystalline and phase purity with no secondary phases is indicated by the crisp and narrow peaks, whereas the high intensity of the (101) peak indicates preferential growth along this plane. The additional peaks are indicative of secondary metabolites that serve as capping and in the *Bauhinia purpurea* extract [43, 44]. The crystalline size of ZnO-NPs was estimated to be 6.3 nm for sol gel method and 27.91 nm for co-precipitation method. The Scherrer equation using the formula given below [45, 46].

2 $$\:D=0.9{\uplambda\:}/{\upbeta\:}\mathrm{c}\mathrm{o}\mathrm{s}{\uptheta\:}$$

The crystallite size (D), the Scherrer constant (K) (about 0.9), the X-ray wavelength ($\:{\uplambda\:}$), the full width at half maximum (FWHM) of the diffraction peak in radians ($\:{\upbeta\:}$), and the Bragg angle ($\:{\uptheta\:}$) are all represented in this equation.

XRD results reveal a highly crystalline hexagonal wurtzite structure with nanometric crystallite size, providing high surface area and structural stability, which facilitate strong adsorption and uniform protective film formation on the CS surface, thereby enhancing corrosion inhibition performance.

### Potentiodynamic polarization measurements

**Fig. 6:**
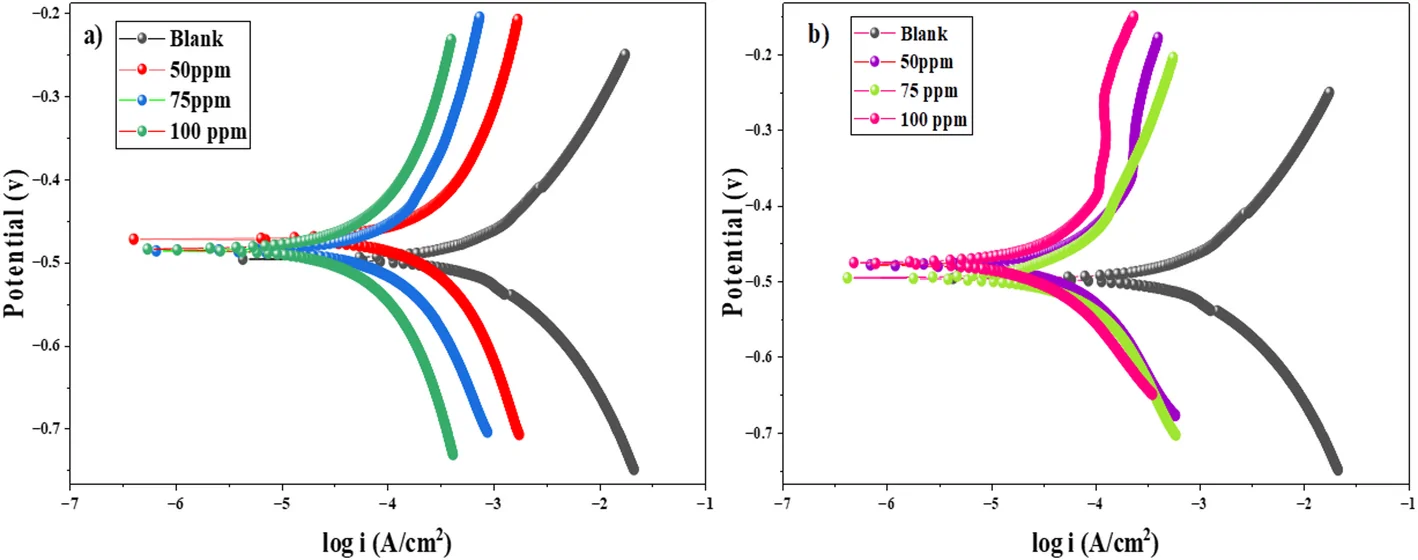
Potentiodynamic polarization curves of CS in 1 M HCl solution in the absence and presence of varying concentrations of ZnO-NPs synthesized by a) co-precipitation method and b) sol–gel method.

The PDP curve of the CS electrode in 1.0 M HCl solutions in the absence and presence of different concentrations of ZnO-NPs at 303 K is displayed in Fig. 6. Electrochemical parameters like corrosion potential (E_corr_), current density (i_corr_), cathodic (β_a_), and anodic (β_c_) slopes are obtained from tafel slope. The findings are presented in Table 2. Two branches make up the plot: the cathodic branch, which represents the hydrogen evolution reaction (reduction of H⁺ ions), and the anodic branch, which shows the dissolving of iron (oxidation). Corrosion potential and current density are direct indicators of the corrosion rate. They can be located by the intersection of both branches [47, 48].

The polarization curves of the blank solution exhibit sharp anodic and cathodic branches with no evidence of passivation, indicating rapid dissolution of the CS surface and accelerated hydrogen evolution. Since the anodic branch did not display a well-defined Tafel region, the linear portion of the cathodic curve was extrapolated to determine the corrosion potential and the corresponding current density [49, 50]. The addition of ZnO-NPs suppressed both anodic and cathodic curves and shifted the polarization curves downward in comparison to the blank. The shift was more evident for the ZnO derived from co-precipitation method as the concentration increased. At 50 ppm, the current density significantly dropped with approximately 75% inhibition, while at 100 ppm, the curves displayed a significant downward displacement with reduced current density to 50 µA cm⁻² and an inhibition efficiency of 94.1%.

At lower concentrations, the inhibitory efficacy of ZnO produced from sol–gel method was higher. The cathodic branch was significantly shifted to lower current densities at 75 ppm, resulting in an efficiency of 94.1%. The entire curve showed a sharp downward displacement at 100 ppm, with an efficiency of 97.7% and current density of 19.4 µAcm⁻^2^. The graph clearly retains its original shape and features in the presence of co-precipitation mediated ZnO-NPs. This suggests that the presence of inhibitors at different doses does not affect the corrosion mechanism of CS in 1M HCl. The cathodic and anodic branch slopes flattened, suggesting that the kinetics of charge transfer were impeded. The development of a dense layer of ZnO-NPs that obstructs active dissolving sites and stabilizes the CS surface against additional attack is suggested by the interesting observation of a partial quasi-passivation plateau with greater inhibitor dosages, especially for sol–gel ZnO. If the difference between the E_corr_ of the blank solution and inhibitor is less than ± 85 mV, the inhibitor may be classified as a mixed-type inhibitor [51, 52].

The ZnO-NPs plots displayed that it was a mixed-type inhibitor, which means that adding ZnO-NPs prolonged the cathodes hydrogen evolution and the anodes dissolution. According to Tafel plots, the polarisation curves with ZnO-NPs added showed a negative shift in the E_corr_ and current density when compared to the blank. This implies that ZnO-NPs formed an adsorption film on the CS that blocked charge transfer on the CS surface, thereby lowering the corrosion rate. Additionally, the cathodic branches structure essentially remained unchanged, indicating that the cathodic corrosion reaction mechanism was unaffected by the addition of ZnO-NPs. It is clear from the anode branch that when ZnO-NPs are added, the curve shifts towards a more negative value, indicating a decreased i_corr_ and corrosion rate. Tafel lines shift toward a reduced i_corr_ in the addition of ZnO-NPs and range in accordance with the inhibitor concentration order. When both inhibitors are present, the current density values decrease, suggesting that the inhibitor molecules have adhered to the CS surface to stop corrosion and increase the inhibitions effectiveness. The blank CS displayed a noticeably higher i_corr_ value of 851.13µA cm^− 2^. The corrosion rate is influenced by the i_corr_ value. The data indicate a superior inhibitory effect of corrosion on CS, with a smaller i_corr_ value indicating a lower corrosion rate. The blank CS consequently displayed the highest rate of corrosion since it corrodes quickly in the HCl media. As the corrosion potential changed to nobler values, the 100 ppm ZnO-NPs exhibited a more pronounced shift towards positive values, according to plots and tabulated data. This suggests that the corrosion rate in these samples decreased. Using Eqs. (3) and (4), respectively, (CR) and inhibition efficiency (IE%) are presented in Table 2.

**Table 2:** Potentiodynamic polarization parameters for the corrosion of CS in 1 M HCl solution in the absence and presence of the inhibitor.

Inhibitor concentration (ppm)	E_corr_ mV/SCE	βc (mV dec^− 1^)	i_corr_ (µA cm^− 2^)	CR mmyr⁻¹	θ	IE%
Blank	− 490	137.7	851.13	10.06	–	–
*Co-precipitation*						
50	− 470	198.1	210	2.48	0.75	75.8
75	− 480	198.4	105	1.24	0.87	87.6
100	− 480	199.6	50	0.59	0.94	94.1
*Sol–gel*						
50	− 470	167.5	233	2.75	0.72	72.6
75	− 470	170	50.4	0.59	0.94	94.1
100	− 480	136.2	19.4	0.22	0.97	97.7

The Tafel cathodic and anodic curves have been shown to shift noticeably as the ZnO-NPs grow. The inhibitor action becomes almost constant as the CS surface has been saturated by the inhibitor molecules. The corrosion rate was shown to decrease as the inhibitor concentration rose.

The corrosion rate and IE Parameters were calculated according to.

3 $${\mathrm{CR}}\;\left( {{\mathrm{mmy}}^{{ - {\mathrm{1}}}} } \right) = \frac{3270\times\:EW\times\:\:{i}_{corr}}{d}$$

4 $$ {\mathrm{Surface}}\;{\mathrm{coverage}}\left( \theta \right) = ~\frac{{i_{{corr}} - {\mathrm{~}}i_{{corr\left( {inhi} \right)}} }}{{i_{{corr}} }} $$

5 $$ {\mathrm{IE}}\% = \frac{{i_{{corr}} - {\mathrm{~}}i_{{corr\left( {inhi} \right)}} }}{{i_{{corr}} }} \times 100 $$

where EW represents the equivalent weight of iron, d is the density of the CS, and $\:{i}_{corr}$ and $\:{i}_{corr\left(inhi\right)}$ are the corrosion current densities in the absence and the presence of an inhibitor, respectively [53, 54].

**Fig. 7:**
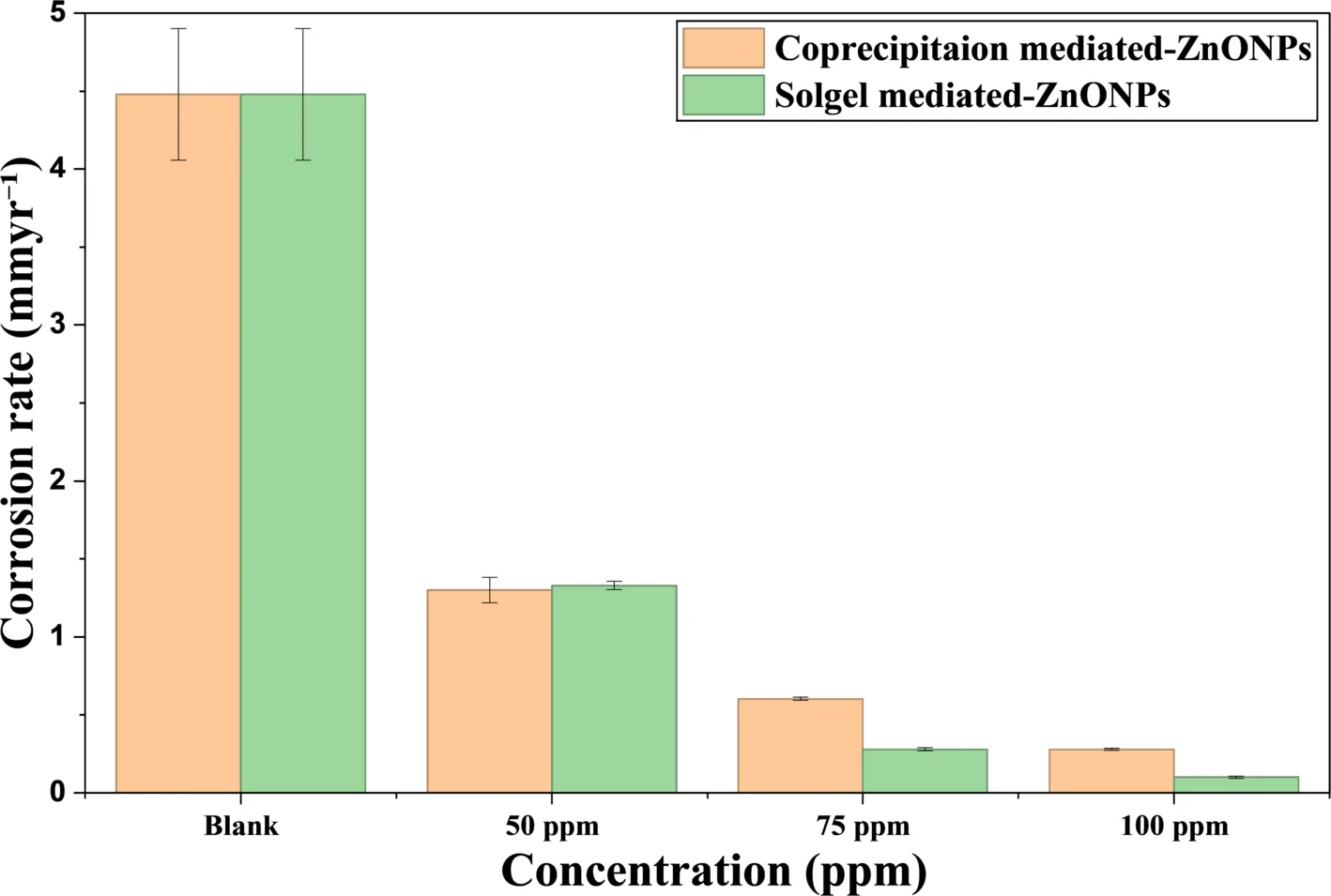
Bar graph illustrating the mean and standard deviation (error bars) of the PDP curve from triplicate measurements.

For both approaches, current density dramatically drops as ZnO-NPs concentration rises. while an 851.13 µA cm^−2^ was obtained in blank solution the value decreased with the addition of ZnO-NPs and eventually to 50 µA cm^− 2^ and 19.4 µA cm^− 2^ for 100 ppm of corrosion inhibitor in the acidic medium. This indicates that the formation of a protective layer on the surface of CS which suppresses the corrosive reaction. The IE of 94.1 and 97.7% for 100 ppm of ZnO-NPs solution, which matches the results of EIS. The addition of an inhibitor results in a decrease in cathodic and anodic slopes in two cases. Table 2 makes it clear that the corrosion rate and current density values obtained for the CS in HCl with ZnO-NPs are lower than the blank. Interestingly, in both cases, the value of these parameters drops off considerably at the lowest tested concentration of corrosion inhibitor [55]. Figure 7 showcases the bar graph illustrating the mean and standard deviation (error bars) of the PDP curve from triplicate measurements.

### Electrochemical impedance spectroscopy

**Fig. 8:**
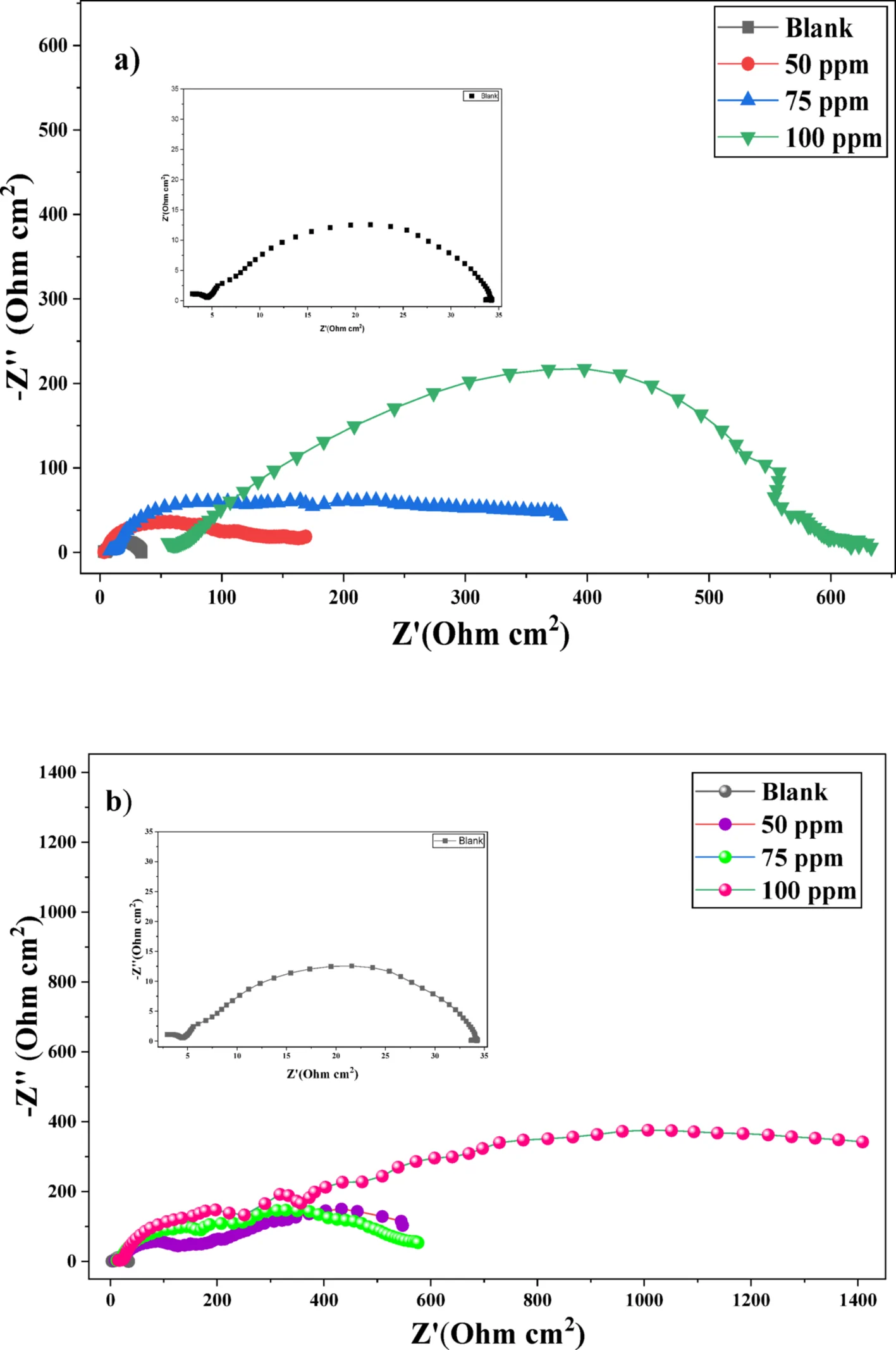
Nyquist plots for CS sample in a 1.0 M HCl solution in absence and presence of ZnO-NPs synthesized by **a)** co-precipitation method and **b)** sol–gel method.

EIS is an electrochemical technique for figuring out the kinetics and features of the corrosion process. Figure 8 displays the Nyquist plots of CS in 1 M HCl at 303 K with and without different concentration of ZnO-NPs, and Table 3 tabulates the corresponding results. There are two loops that define the Nyquist plot. A huge secondary capacitive loop in the low frequency (LF) region comes after the initial loop, which is a capacitive loop in the higher frequency (HF) region. The charge transfer resistance is represented by the primary capacitive loop. Each panel shows a single semicircle, indicating that the inhibitory extract molecules in the HCl had no effect on the single charge transfer process that occurred during the anodic dissolution of CS [56] .

**Fig. 9:**
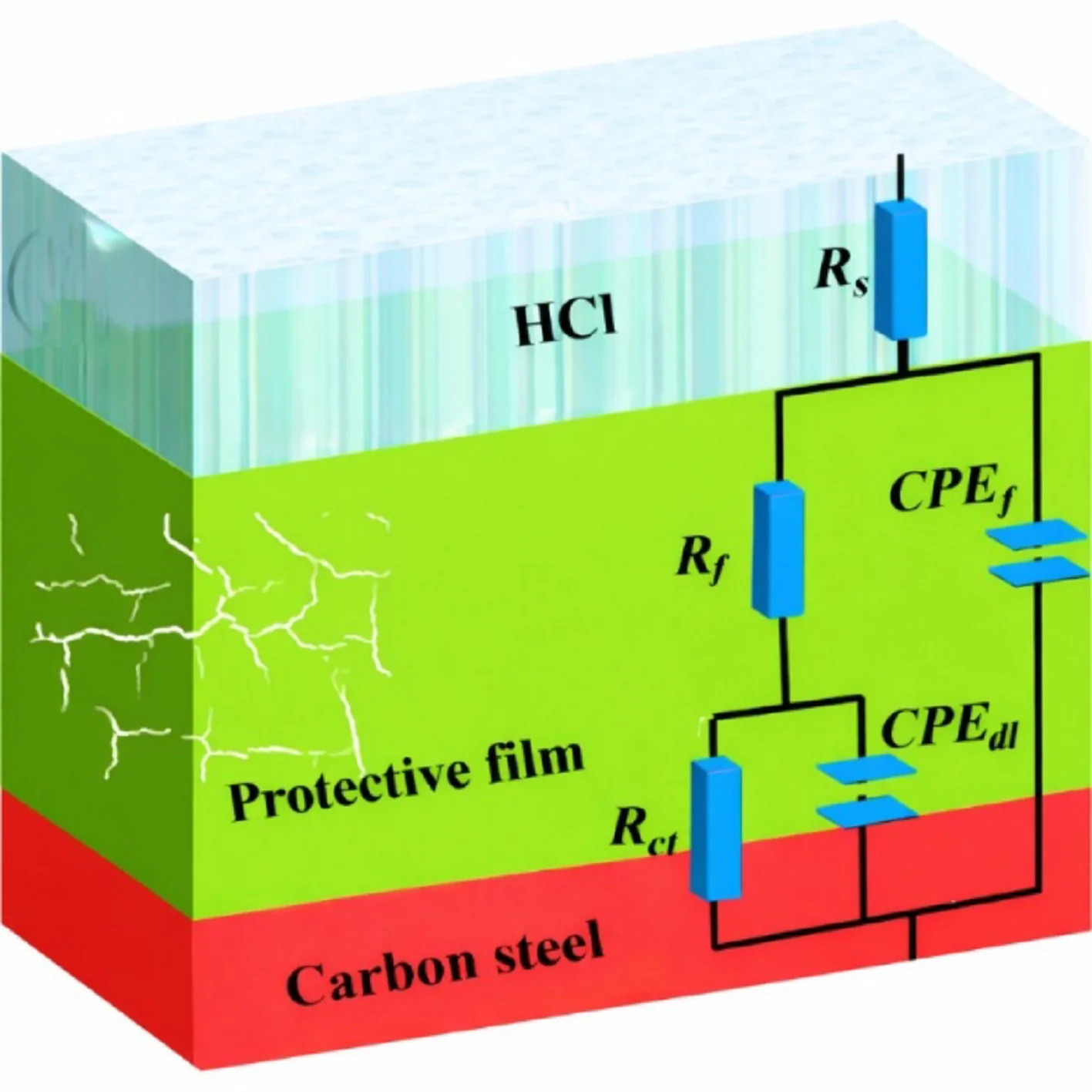
The equivalent circuit used to analyse the impedance data.

A modest diameter semicircle is visible in the blank solution, as indicated by the electrochemical impedance diagram, and its size grows as the inhibitor and concentration levels rise. This suggests that the corrosion mechanism did not alter despite the presence of the inhibitor. Increasing ZnO-NPs concentration results in the development of a denser protective layer, which enhances capacitive resistance values and hinders the charge transfer between CS and solution. This is due to the quick adsorption of ZnO-NPs to the surface of the CS electrode, forming an adsorption film at the vicinity between the CS surface and the solution that prevents Fe from dissolving and slows down the corrosion rate.

The impedance modulus rises from high to mid frequencies and stabilises beyond that, indicating that the mid-frequency range reflects the overall corrosion process. At this frequency range, a higher impedance modulus is associated with more effective corrosion inhibition. The high-frequency (HF) capacitive loop corresponds to charge transfer at the film-solution interface. The formation of a protective layer on the metallic surface is correlated with the capacitive loop observed at low frequency (LF). The barrier film prevents the metal from corroding and limits direct contact with aggressive environments. The increase in diameter is accountable to the growth of a barrier layer on the CS surface against corrosive electrolyte, which improves the resistance of CS bars to degradation. The figure shows that as the concentration gradient of ZnO-NPs increases, the impedance values at intermediate frequencies increase accordingly. This proportional rise in impedance with ZnO-NPs concentration at mid frequencies indicates that adsorption of the inhibitor species on the CS surface effectively suppresses the charge transfer process [57–61].

Table 3 demonstrates that even at low concentrations, the corrosion rate dramatically dropped in comparison to the blank solution, and that the charge transfer resistance (R_CT_ ), and solution resistance (R_S_), double layer capacity (C_dl_), all progressively rose as the inhibitor concentration increased. This suggests that CS corrosion is considerably impacted by the inhibitor [62]. The Nyquist semicircle of the secondary capacitance loop enlarged as the concentration rose. Like the impedance modulus, the phase angle also increases as the inhibitor concentration increases, reaching about 50° at 100 ppm. This again suggests that a protective layer is forming because of the more capacitive behaviour [63, 64].

**Fig. 10:**
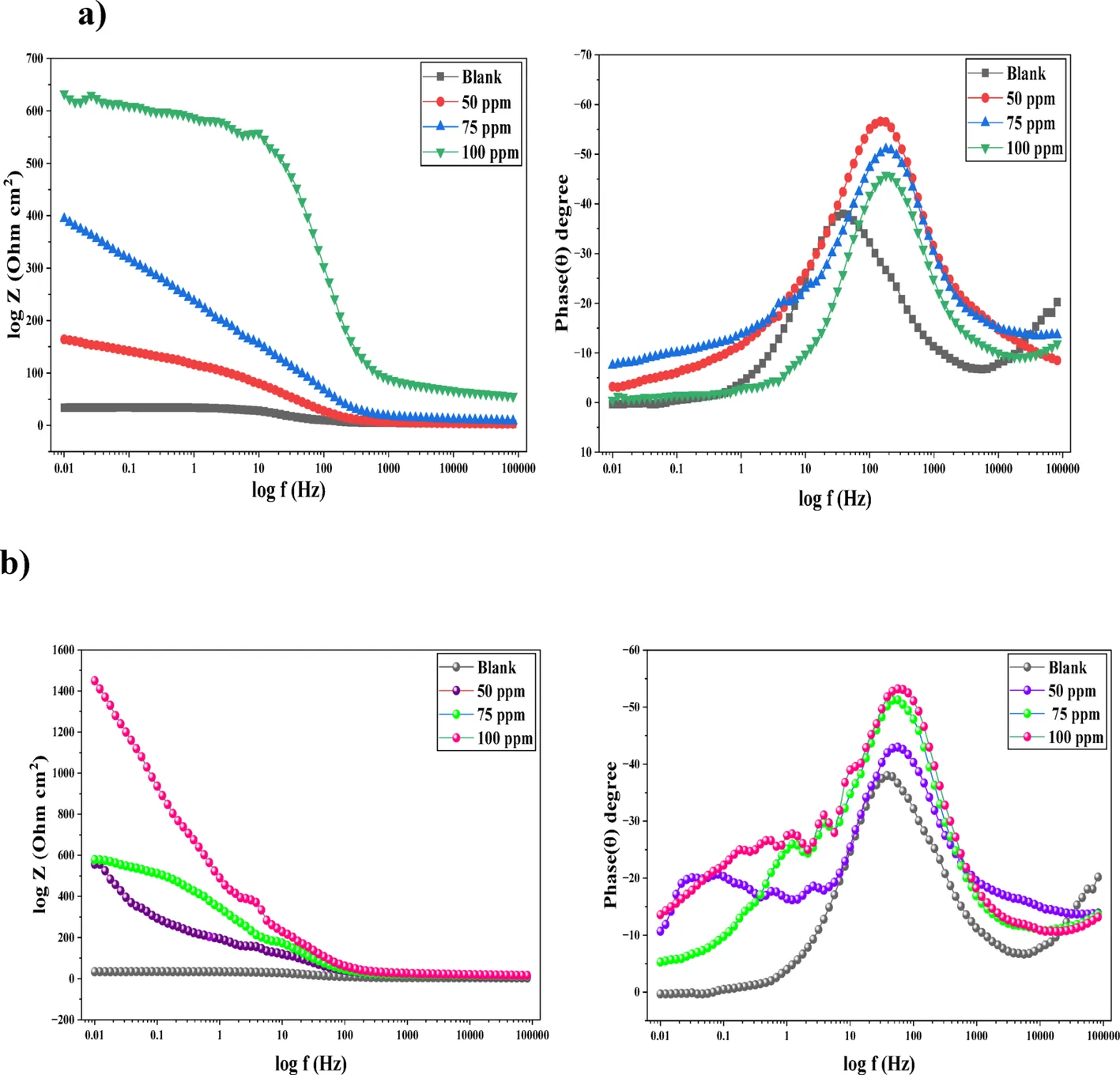
Bode plots of CS in 1 M HCl with and without varying concentrations of **a)** Co-precipitation mediated-ZnO-NPs. **b)** Solgel mediated-ZnO-NPs.

A suitable five element circuit was fitted by using ZSimpWin software where the elements are R_S_, R_CT_, R_F_ (film resistance), and due to the heterogeneity of the metal surface two CPE (constant phase elements) are used instead of as shown in Fig. 9 [65]. In comparison to the resistance provided by the primary capacitive loop (R_CT_) and secondary capacitive loop (R_F_), the R_S_ is a very small figure Total polarization resistance, or R_P_, is calculated using the formula.

6 $$ R_{P} = R_{{CT}} + R_{F} $$

  

Consequently, the percentage inhibition was determined using the equations.

7 $$IE{{\% }} = \frac{{R_{{P\left( {inh} \right)}} - R_{P} ~}}{{R_{{P\left( {inh} \right)}} }}~ $$

Equation (8) was implemented to determine the double-layer capacitance and the CPE impedance.

8 $$ C_{{dl}} = Q_{{dl}} (\omega _{{max}} )^{{n - 1}} $$

C_dl_ is the double-layer capacitance, ω_max_ is the frequency at which the imaginary impedance (Z) is at its maximum, Y_o_ is the CPE constant, and n is the CPE exponent, which denotes the unevenness of the electrode surface. In the case of *n* = 1, the CPE functions similarly to a perfect capacitor [66].

**Table 3:** EIS data for CS corrosion in a 1.0 M HCl solution with and without ZnO-NPs.

Inhibitor concentration (ppm)	*R*_S_ (Ωcm^2^)	*R*_F_ (Ωcm^2^)	*R*_CT_ (Ωcm^2^)	*R*_*P*_ (Ωcm^2^)	*n*	C_dl_ (µFcm^− 2^)	χ^2^ × 10^− 3^	IE%
Blank	1.22	2.47	23.36	26.13	0.83	359	1.41	–
*Co-precipitation*								
50	2.88	5.66	95.54	101.2	1	113.7	2.55	74
75	5.50	67.57	269.9	337.47	0.42	98	3.57	92
100	28.28	1615	1045	2660	0.98	2.34	1.08	99
*Sol–gel*								
50	2.23	3.52	411.8	415.32	0.42	40.10	3.31	93
75	11.12	66.66	413.9	480.56	0.56	57.50	6.76	94
100	12.30	111.4	1267	1378.4	0.52	30.50	7.67	98

Table 3 clearly shows that the values of R_S_, R_CT_, R_F_, and R_P_ for the solutions containing 1 M HCl and ZnO-NPs are higher than those for the control solution. Further analysis of Table 4 reveals that the greatest inhibition [67].

The observed increase in $\:{R}_{s}$ from the blank to 100 ppm is because the inhibitor itself alters the electrochemical environment: at higher concentration, the molecules may adsorb on the metal surface, create a partially resistive interfacial layer, reduce ion mobility, or slightly change the effective conductivity of the solution. The variation results from the concentration-dependent effect of the inhibitor on the solution-interfacial region rather than as a change in the experimental setup.

The charge transfer resistance increases, and the double layer capacitance decreases with increasing inhibitor concentration, as seen by the EIS data, showing increased inhibition efficiency. C_dl_ denotes the charge stored at the metal-solution interface, which directly influences charge transfer between the metal and the solution [68]. A drop in capacitance shows that a protective layer has formed, blocking the attack of H^+^ and Cl^−^ ions on the CS surface. The highest C_dl_ (359 µF cm^−2^) and lowest R_CT_ (23.36 Ω cm^2^) for the blank sample both indicates significant corrosion. On the other hand, ZnO-NPs produced using sol–gel and co-precipitation methods both greatly enhance protection, with efficacy increasing gradually from 50 to 100 ppm. The co-precipitation approach performs even better with 99% efficiency and higher overall resistance, indicating its superior shielding capacity, whereas the sol–gel method obtains an inhibition efficiency of 98% with a rapid decline in C_dl_ (30.5 µFcm^−2^) at 100 ppm [19, 69, 70]. Hence, both EIS and PDP data match with each other, mediated ZnO-NPs can be chosen as an effective corrosion inhibitor because even at a small concentration, the inhibitor effectively retards corrosion [71, 72]. The adsorption behaviour of ZnO-NPs on the CS surface was further evaluated using Bode plots, as shown in Fig. 10. Compared to the blank solution and other concentrations, the impedance modulus significantly increased upon the addition of 100 ppm of ZnO-NPs. This enhancement in impedance indicates improved surface protection and confirms that 100 ppm provides superior corrosion resistance compared to the other tested concentrations.

The apparent difference in the relative performance of the co-precipitation and sol–gel synthesized ZnO-NPs obtained from PDP and EIS measurements arises primarily from the different electrochemical principles of the two techniques rather than inconsistencies in the experimental results. PDP involves the application of an external potential that perturbs the electrode surface and may partially disrupt the adsorbed inhibitor film, thereby reflecting the kinetics of the anodic and cathodic reactions under polarized conditions. In contrast, EIS is performed close to the OCP using a small AC perturbation, providing information on the stability, integrity, and barrier properties of the protective film under near-equilibrium conditions. Consequently, EIS generally reports slightly higher inhibition efficiencies when the inhibitor forms a stable and compact adsorbed layer. In the present study, although the relative ranking differs slightly between the two techniques, both consistently demonstrate excellent corrosion protection with inhibition efficiencies exceeding 94% at the optimum concentration. The small variation is therefore attributed to the inherent differences between the electrochemical techniques and the characteristics of the protective films under their respective measurement conditions, rather than to experimental inconsistencies [73, 74].

### Atomic absorption spectroscopy (AAS)

Atomic absorption spectroscopy is used to determine the extent of iron dissolution from CS after 24 h immersion in 1 MHCl solution in the absence and presence of the inhibitor at different concentrations. The blank solution exhibited the highest Fe concentration indicating severe corrosion of the metal surface in the acidic medium. Upon addition of the inhibitor, a significant reduction in Fe dissolution was observed, confirming the formation of a protective adsorbed film on the CS surface [75].

**Table 4:** AAS data for CS corrosion in a 1.0 M HCl solution with and without ZnO-NPs.

Concentration (ppm)	Concentration of Iron dissolved (mg L^−1^) Co-precipitation	Concentration of Iron dissolved (mg L^−1^) Sol–gel
Blank	3.45	3.45
50	1.21	1.46
75	1.06	1.39
100	0.83	0.73

The AAS results are in good agreement with the electrochemical measurements, thereby confirming the concentration-dependent corrosion inhibition behaviour of the inhibitor are given in Table 4 [76].

### Scanning electron microscope (SEM)

The topography and surface morphology of synthesized ZnO-NPs were examined using SEM, as shown in Fig. 8. Following the validation of the XRD results, the sample was further prepared for the SEM analysis. The majority of ZnO-NPs were found to have a spherical, hexagonal, rod-like form. Additionally, as is common with green synthesis NPs, the ZnO-NPs exhibit a minor agglomeration. This is because biosynthesised NPs have a larger surface area and agglomeration is caused by their long-lasting attraction for one another, which may have resulted from the capping action of the leaf extracts phytochemicals [77, 78].

**Fig. 11:**
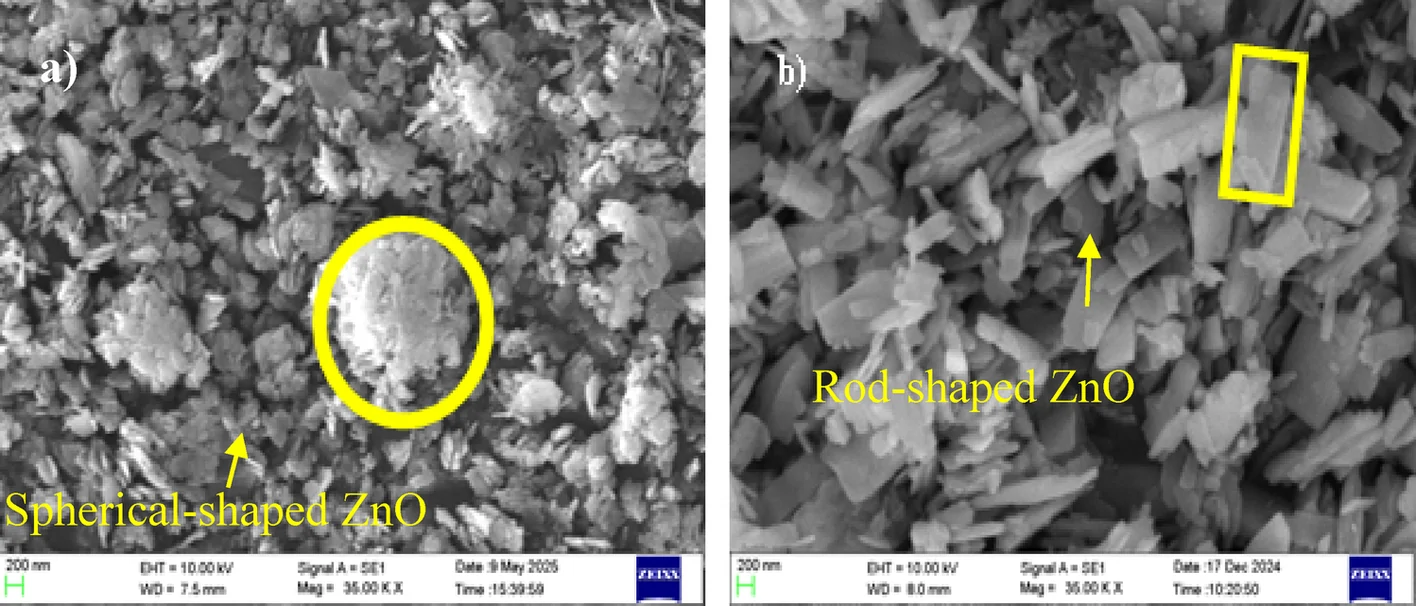
Schematic illustration of SEM of ZnO-NPs synthesised using the a) co-precipitation method and **b)** sol–gel method.

Figure 11a shows spherical particles of ZnO synthesised from the co-precipitation method, while Fig. 11b shows rod-shaped ZnO from the sol–gel approach [79, 80]. Particles seem to be agglomerated, and some individual hexagonal crystals are clearly visible. SEM images appear at magnifications of 200 nm.

**Fig. 12:**
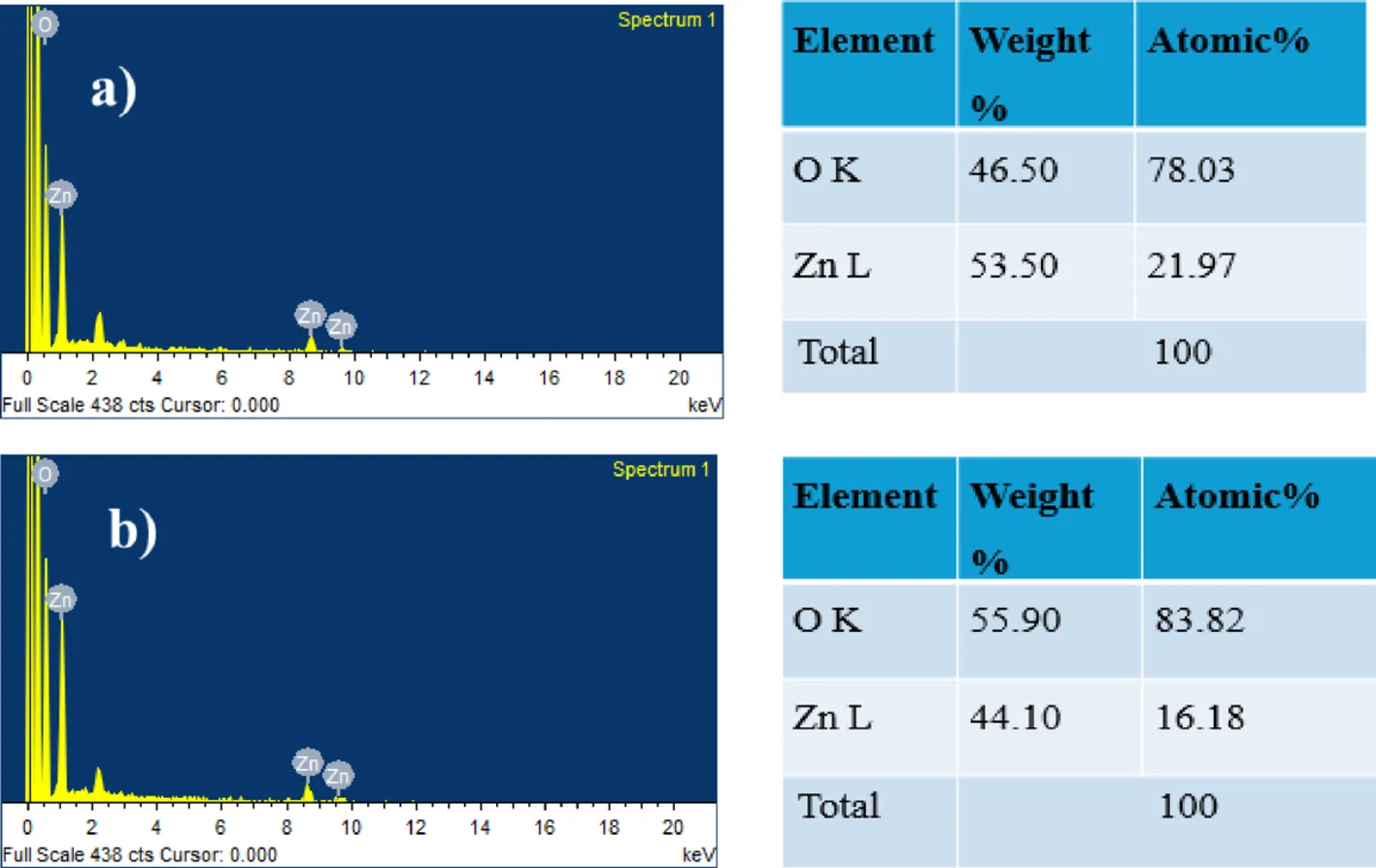
EDS spectra of ZnO-NPs synthesised using **a) co**-precipitation method and **b) sol**–gel method.

The basic elemental composition of the green synthesised ZnO-NPs is validated by the EDX study is displayed in Fig. 12. The results showed that only Zn and O elements were present, indicating high purity. The EDX spectra of sol–gel mediated Zn supplied 44.10 wt% (16.18 at%) and O contributed 55.90 wt% (83.82 at%), which closely matched the stoichiometry of ZnO. Zn and O were detected in the spectrum of co-precipitation mediated ZnO-NPs with Zn at 53.50% (21.97 at%) and O at 46.50 weight% (78.03 at%). This suggests that ZnO-NPs was successfully formed with high purity and close to stoichiometric ratios. The optical absorption peak at 1 keV in the prepared sample indicated the presence of Zn. These findings provide more evidence of the green synthesis efficacy, as the plant extracts bioactive components help stabilise and reduce Zn ions while also avoiding contamination during synthesis.

**Fig. 13:**
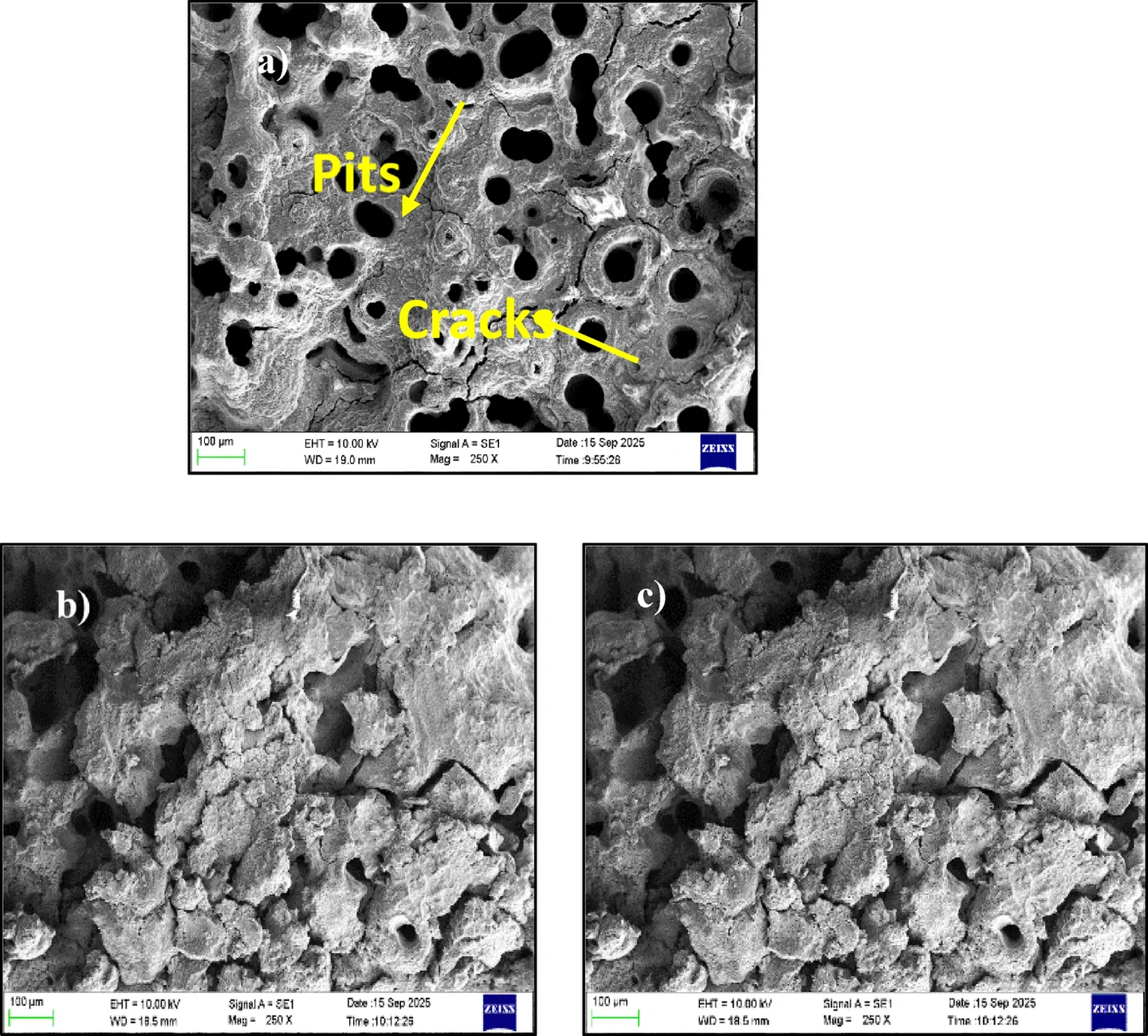
SEM micrographs of CS samples after 3 days of immersion period in 1M HCl a) without ZnO-NPs b**)** with ZnO-NPs from the co-precipitation method **c) **with synthesised ZnO-NPs from solgel-method.

SEM topography images and their corresponding EDS of CS surfaces following three days of immersion in HCl, both devoid and in the presence of 1 M HCl, are discussed below in Fig. 13. The finely polished CS surface, which is smooth and free of defects, is allowed to contact 1M HCl [81].

Figure 13a shows that CS in the blank solution is extremely corrosive because it causes surface cracks and pits in addition to scratches; however, corrosion rate is decreased when an inhibitor is added, resulting in fewer surface pits and cracks and fewer cracks. A protective layer developed on the CS surface as a result of deposits that were also seen here [82].

**Fig. 14:**
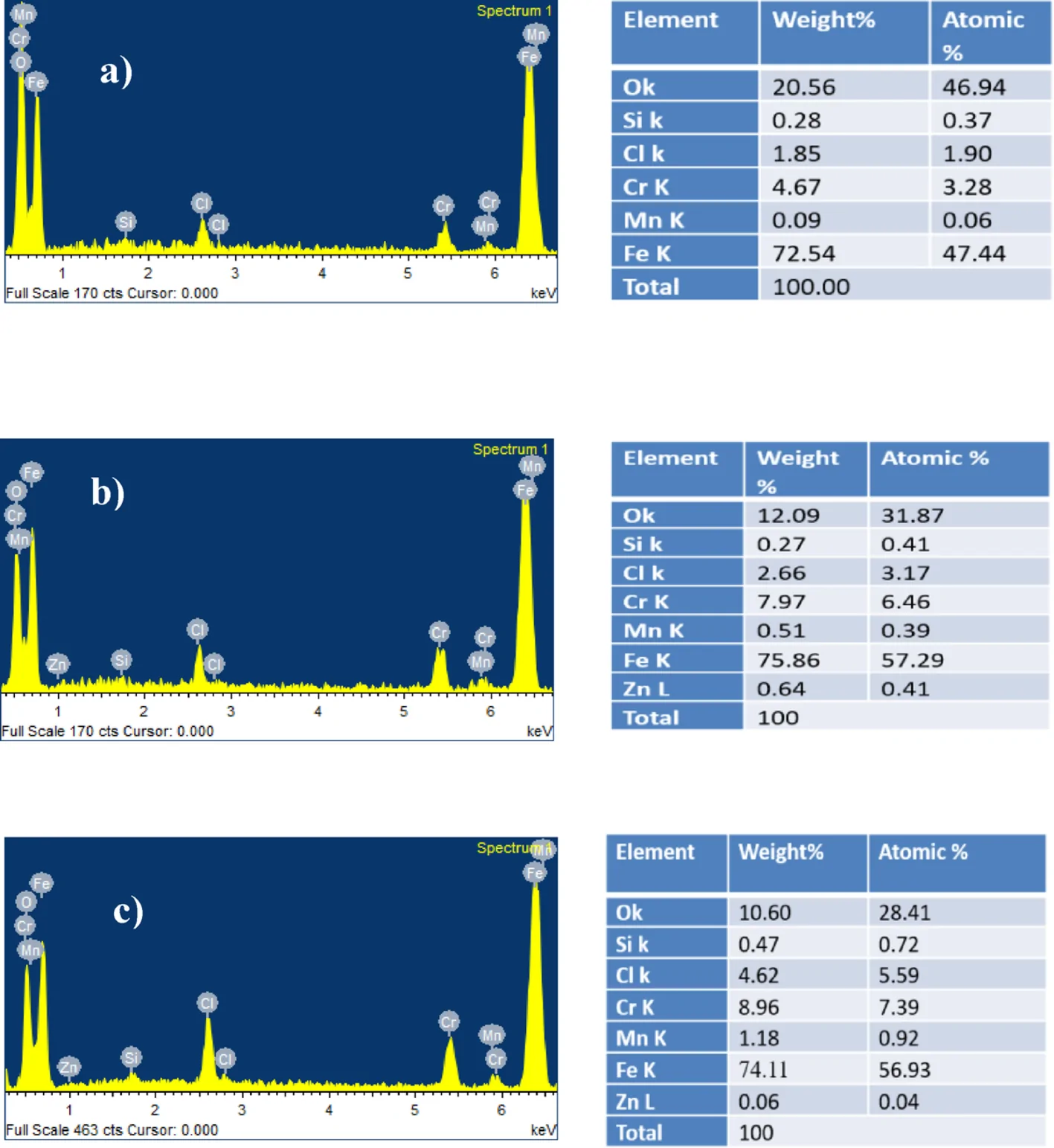
EDS spectra of CS immersed in 1 M HCl a) without ZnO-NPs b) with ZnO-NPs synthesised using co-precipitation method and c) with CS immersed in ZnO-NPs synthesised using sol-gel method.

The EDS data in Fig. 14 for CS immersed in 1M HCl clearly show that ZnO-NPs act as an effective corrosion inhibitor by forming a protective surface film. In the uninhibited samples, a high iron content (72.54 wt%) along with significant oxygen (20.56 wt%) and chlorine (4.67 wt%) indicates severe corrosion and chloride ion adsorption causing surface degradation. When ZnO-NPs are added, chlorine content decreases markedly (1.8–2.6 wt%), while Zn (0.06–0.64 wt%) and oxygen increase moderately (12 wt%), reflecting the presence of a stable ZnO-based barrier layer. This layer reduces the penetration of corrosive chloride ions and limits Fe dissolution, protecting the CS surface.

Alloying elements such as chromium, manganese, and silicon remain consistent across samples, confirming uniform alloy chemistry. Overall, the EDS evidence supports ZnO-NPs as a powerful inhibitor that significantly enhance CS resistance in acidic media by reducing corrosion product formation and chloride attack, consistent with reported literature on ZnO-NPs inhibition in acidic environments [83].

### XPS analysis

**Fig. 15:**
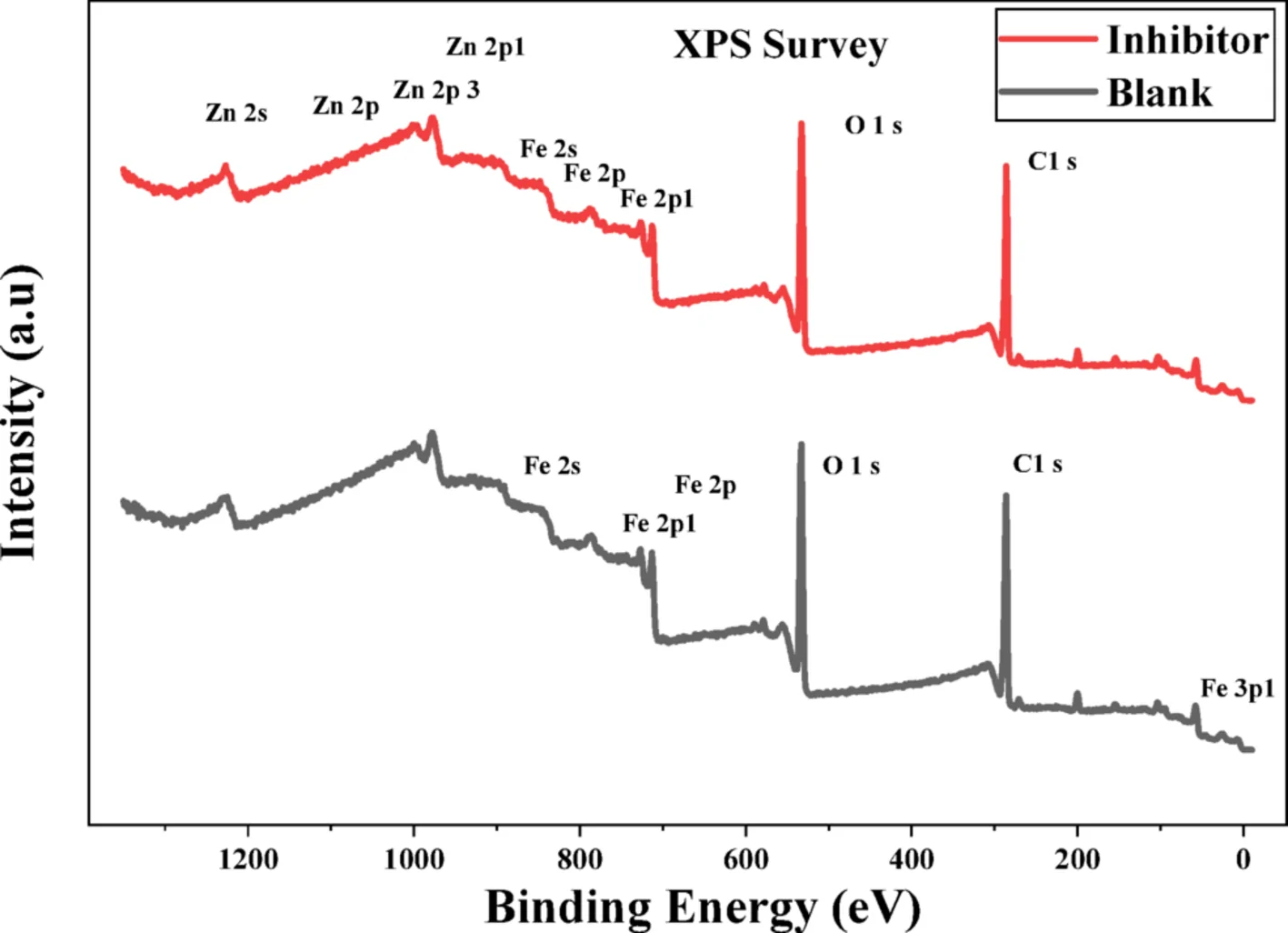
XPS survey graph of CS with and without ZnO-NPs.

**Fig. 16:**
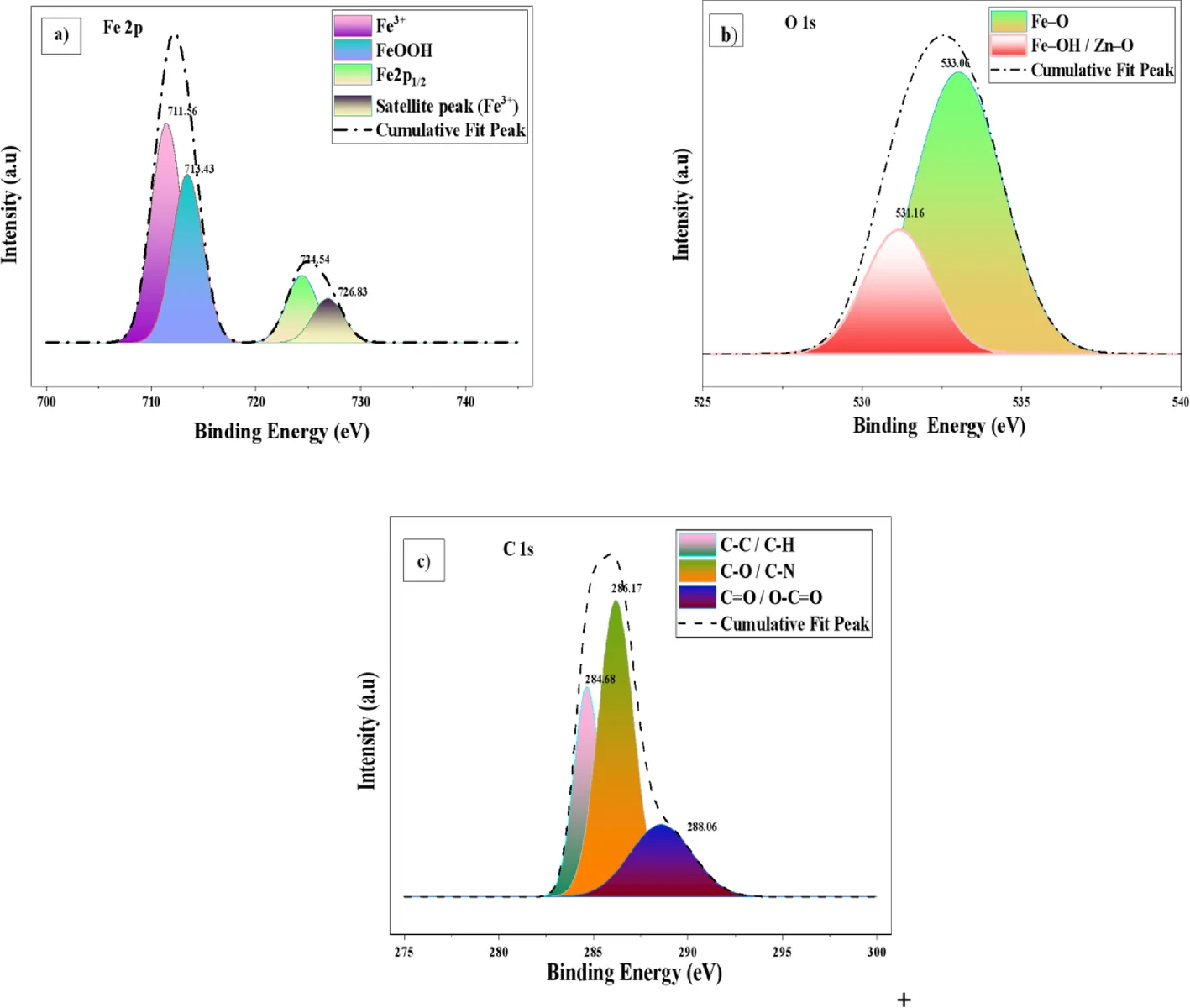

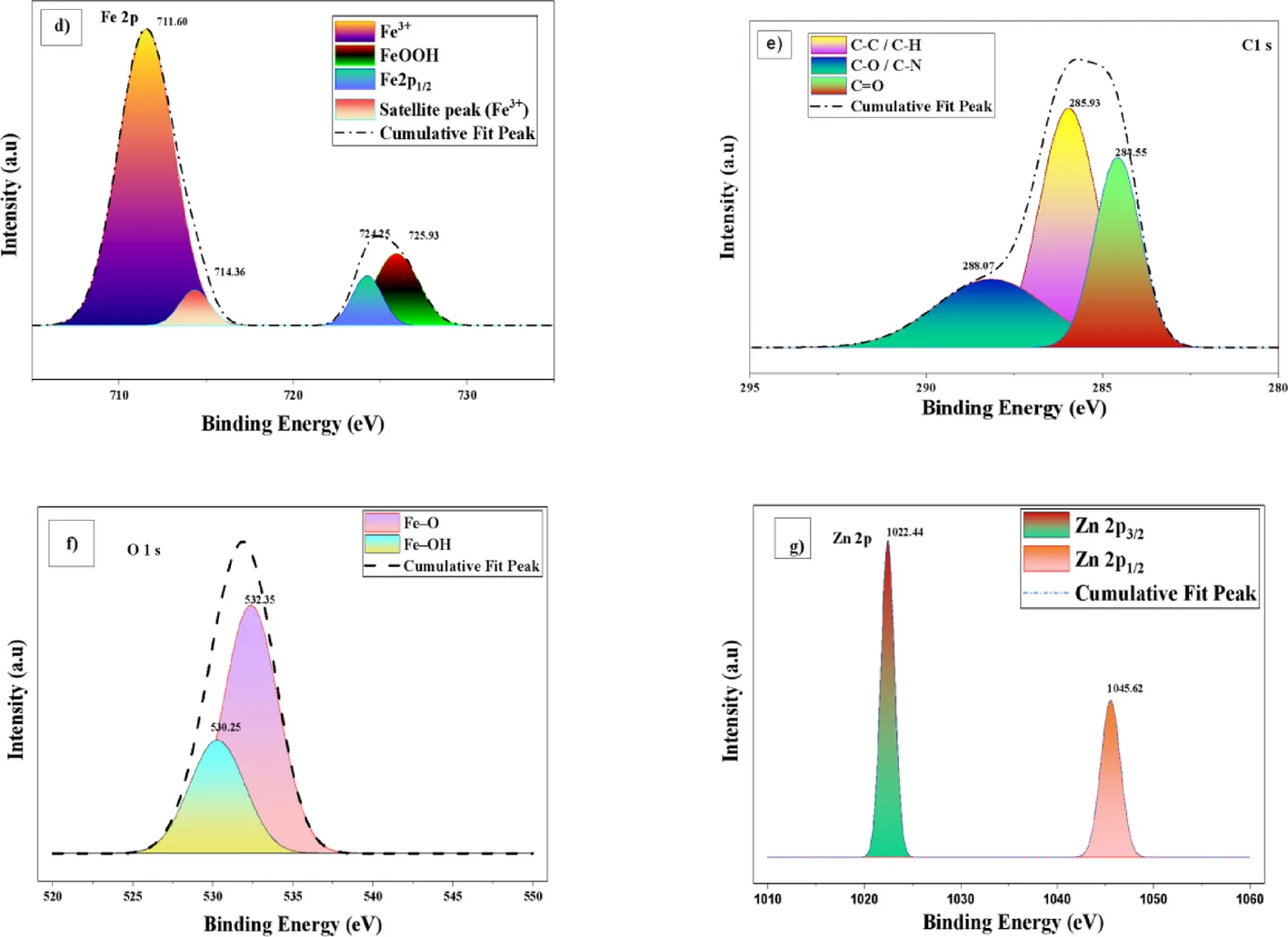
XPS survey graph of CS in 1M HCl a-c) without ZnO-NPs **d**-**g** ) with ZnO-NPs.

The surface chemical composition and inhibition mechanism of CS in 1M HCl in the absence and presence of inhibitor were investigated using XPS given in Fig. 15 and 16 [84]. In the blank system, Fe 2*p* spectra showed peaks at 710.8 eV and 724.5 eV. These matched Fe 2*p*_3/2_ and Fe 2*p*_1/2_ spin-orbit components. Satellite peaks appeared prominently. Fe^3+^ states formed, including Fe_2_O_3_ and FeOOH species. These results indicate the formation of adherent and porous oxide layer on the CS surface [85]. A peak dominates the O 1s spectrum at 529.8 eV attributed to lattice oxygen (Fe–O), along with higher binding energy components (531–533 eV) corresponding to hydroxyl species and adsorbed water, further supporting extensive surface oxidation. The C 1*s* spectrum shows a dominant peak at 284.8 eV attributed to adventitious carbon (C–C/C–H), which is commonly associated with surface contamination and does not significantly contribute to corrosion protection [86, 87].

In contrast, the inhibited system shows significant modifications in surface chemistry. The Fe 2*p* peak intensity decreases and exhibits a minor shift toward lower binding energy, signaling reduced oxidation and suppression of iron dissolution due to inhib

itor adsorption. The O1*s* spectrum shows enhanced contributions from hydroxyl and adsorbed oxygen species, suggesting the formation of a more stable and compact hydroxylated layer.

The C 1*s* spectrum reveals additional components at higher binding energies corresponding to C–O and O–C=O groups, confirming the adsorption of organic moieties from the inhibitor on the CS surface. Notably, distinct Zn 2*p* peaks observed at 1021.8 eV (Zn 2*p*_3/2_) and 1044.9 eV (Zn 2*p*_1/2_) confirm the incorporation of ZnO-NPs into the protective film. The combined presence of iron oxides/hydroxides, adsorbed organic species, and ZnO-NPs leads to the formation of a compact, adherent barrier layer, effectively blocking active corrosion sites. This suggests that the inhibition mechanism involves synergistic interaction between ZnO-NPs and organic constituents through adsorption and surface complexation, resulting in enhanced corrosion resistance of CS [88].

### Adsorption studies

**Fig. 17:**
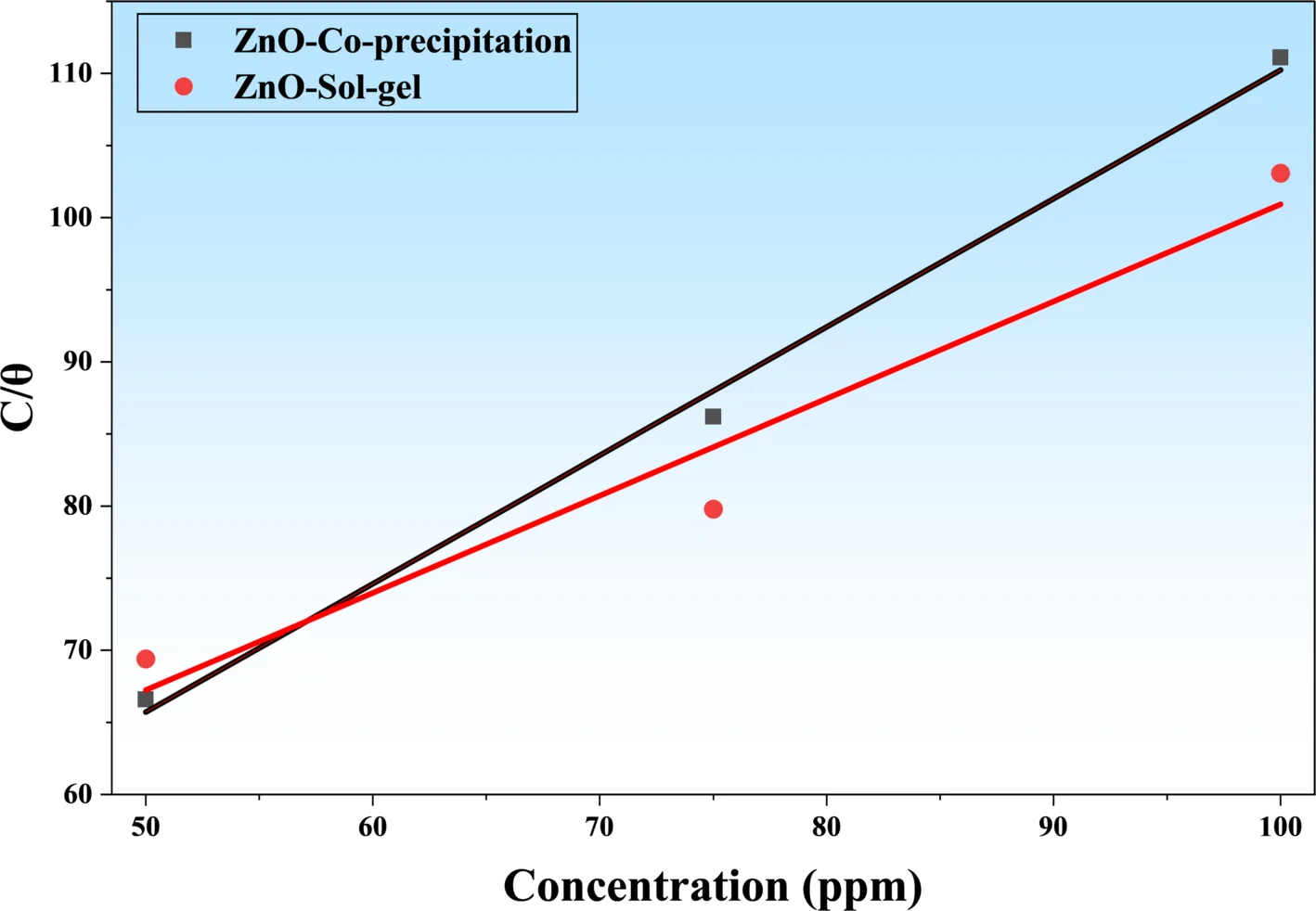
Langmuir adsorption isotherm for the adsorption of ZnO-NPs onto the CS surface in 1 M HCl.

To explore the adsorption behaviour of ZnO-NPs at CS and HCl solution interface, various isotherm models such as Frumkin, Flory-Huggins, Langmuir, El-Awady, and Temkin were evaluated using surface coverage (θ) values derived from PDP data. Among the models tested, the Langmuir adsorption isotherm provided the best linear correlation, which is defined by Eq. 9. The Langmuir model assumes that adsorption occurs on a uniform surface with identical active sites, that there is no lateral interaction between the adsorbed species, and that at maximum adsorption a monolayer is formed. The surface coverage (θ) values as a function of inhibitor concentration were derived from PDP data, Fig. 17.

In this model, C_inh_ represents the molar concentration of the tested inhibitors, and θ corresponds to the surface coverage, which was determined from electrochemical measurements. A linear relationship was obtained when plotting C against C/θ, producing a straight line with both slope and correlation coefficient values close to unity.

9 $${\mathrm{Langmuir}}\frac{{C_{{inh}} }}{{\theta  }} = \frac{1}{{{\mathrm{k}}}} + C_{{inh}} $$

  

For the co-precipitation-derived ZnO-NPs, a linear relationship was obtained with a correlation coefficient (R²) of 0.995, and the slope (0.8902) was close to unity, confirming good agreement with the Langmuir model [89].

**Fig. 18:**
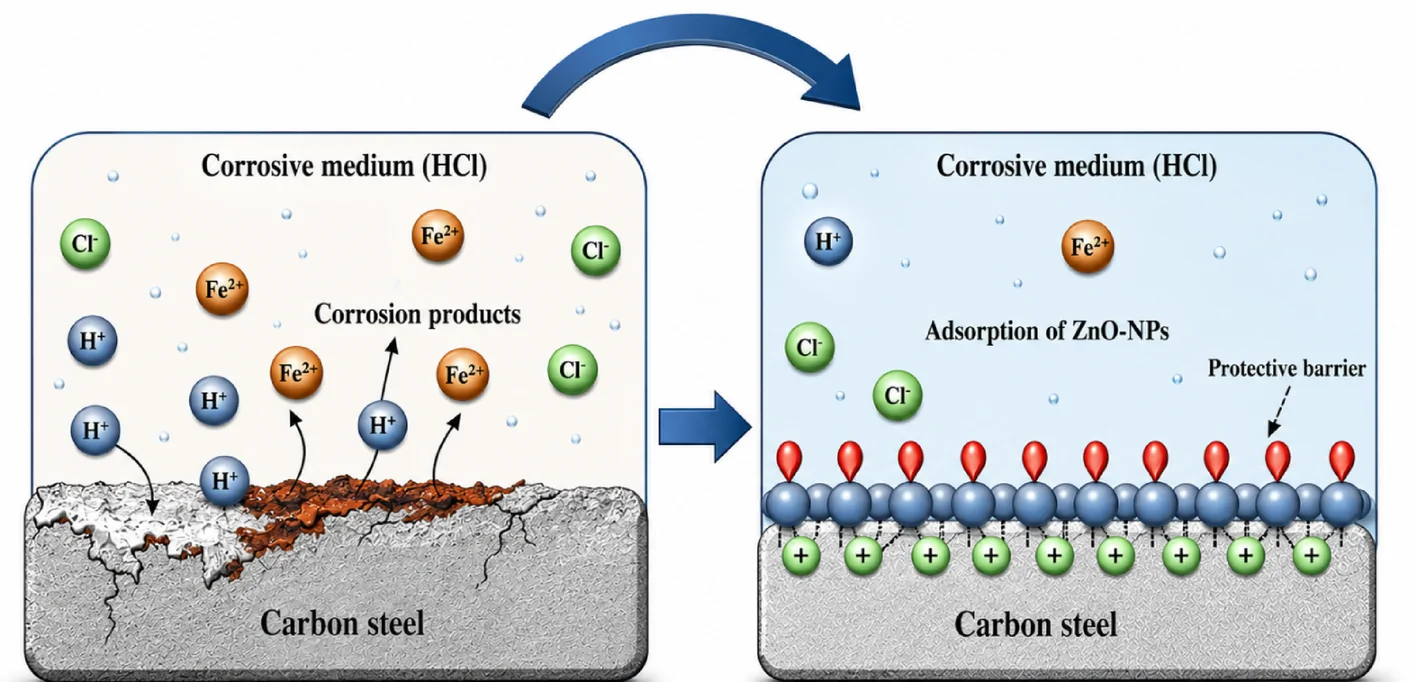
Schematic representation of the mechanism of ZnO-NPs as corrosion inhibitor on CS.

In the HCl medium, ZnO-NPs are transported toward the CS surface, where anodic and cathodic reactions occur simultaneously. The anodic reaction involves the oxidation of iron, producing Fe^2+^ ions, while the cathodic reaction consists of the reduction of H⁺ ions, accompanied by the release of hydrogen gas.

10 $$Fe\to\:{Fe}^{2+}+2{e}^{-} \;\;\left( {{\text{anodic reaction}}} \right) $$

11 $$ 2{\mathrm{H}}^{ + } + {\mathrm{2e}}^{ - } \to {\mathrm{H}}_{2} \;\;\left( {{\text{cathodic reaction}}} \right) $$

Furthermore, the dissolved oxygen in an HCl solution exposed to the air reduces to water in the manner described below:

12 $$ {\mathrm{O}}_{2} + 4{\mathrm{H}}^{ + } + 4~{\mathrm{e}}^{ - } \to 2{\mathrm{H}}_{2} {\mathrm{O}}\;\;\left( {{\text{cathodic reaction}}} \right)~ $$

From the experimental studies, the corrosion inhibition mechanisms of the ZnO-NPs are proposed, as illustrated in Fig. 17. Both physical and chemical processes lead to the adsorption of inhibitor compounds on the CS surface. Physical adsorption arises from electrostatic interactions between deposited electrons on the CS surface and the inhibitor species. In addition, chemical adsorption is promoted by heteroatoms possessing lone electron pairs, which enhance binding with the CS surface. Charge transfer from the d-orbitals of Fe to the non-bonding π orbitals of the inhibitor molecules is facilitated by the increasing negative surface potential of CS caused by the accumulation of electrons. A protective barrier that drastically decreases corrosion is created as a result of this synergistic adsorption process [90].

The ZnO-NPs primarily interact with iron sites on the steel surface, forming an oxide layer. Heteroatoms (O, N, and S), hydroxyl and amino groups, and unsaturated bonds (− C=C−, −C=O− and aromatic ring) in inhibitor molecules can donate free electrons to the empty d-orbital of iron atoms to form coordination bonds and receive electrons from iron with antibond orbitals to form a feedback bond. ZnO-NPs tend to adsorb parallel to the CS surface, facilitating the formation of an effective protective layer. ZnO-NPs effectively obstruct the direct contact between Cl and the CS, thereby improving their corrosion resistance [91, 92].

## Conclusion

The *Bauhinia purpurea* mediated ZnO-NPs effectively protect CS in 1 M HCl by forming an adsorbed barrier film. The system behaves as a mixed-type inhibitor, as confirmed by electrochemical measurements. ZnO-NPs synthesized by sol–gel and co-precipitation routes showed excellent corrosion resistance, with maximum inhibition efficiencies of about 94% and 97% at 100 ppm and 303 K, respectively, as obtained from potentiodynamic polarization measurements. In line with these findings, electrochemical impedance spectroscopy indicated even higher inhibition performance, with efficiencies of approximately 99% for the co-precipitation-derived ZnO-NPs and 98% for the sol–gel derived ZnO-NPs at the same concentration. Electrochemical impedance spectroscopy measurements showed a significant increase in charge transfer resistance and polarisation resistance upon the addition of ZnO-NPs, further confirming its effectiveness in forming a protective film on the metal surface followed by Langmuir adsorption isotherm. Surface analyses (SEM, EDS, XPS) reveal a smoother, more uniformly covered steel surface in the inhibited solution compared with the severely attacked uninhibited sample. FTIR, and EDS evidences the adsorption of phytochemical and ZnO-NPs on the metal, consistent with the increased polarization resistance and the formation of a protective organic-inorganic layer on CS. Overall the system provides an effective ecofriendly and low-cost alternative to conventional synthetic inhibitors.

Compared with previously reported green ZnO-based corrosion inhibitors, the *Bauhinia purpurea*-mediated ZnO-NPs exhibited comparable or superior corrosion protection while demonstrating the influence of synthesis route on inhibition performance. The developed system offers an efficient, eco-friendly, and cost-effective alternative to conventional synthetic inhibitors. Future research should focus on assessing the long-term stability of the protective film under practical service conditions and investigating the antibacterial properties of ZnO-NPs for simultaneous corrosion and biofouling mitigation.

## Data Availability

Data supporting this study is available from the corresponding author upon reasonable request.
